# Widespread Alternative Splicing Changes in Metastatic Breast Cancer Cells

**DOI:** 10.3390/cells10040858

**Published:** 2021-04-09

**Authors:** Jagyeong Oh, Davide Pradella, Changwei Shao, Hairi Li, Namjeong Choi, Jiyeon Ha, Sonia Ruggiero, Xiang-Dong Fu, Xuexiu Zheng, Claudia Ghigna, Haihong Shen

**Affiliations:** 1 School of Life Sciences, Gwangju Institute of Science and Technology, Gwangju 500-712, Korea; jgoh@gist.ac.kr (J.O.); njchoi@gist.ac.kr (N.C.); hajiyn@gist.ac.kr (J.H.); xuexiuzheng@gist.ac.kr (X.Z.); 2 Institute of Molecular Genetics “Luigi Luca Cavalli-Sforza”, National Research Council, Via Abbiategrasso 207, 27100 Pavia, Italy; davide.pradella@igm.cnr.it (D.P.); sonia.ruggiero01@universitadipavia.it (S.R.); 3 Department of Cellular and Molecular Medicine, University of California, San Diego, La Jolla, CA 92093-0021, USA; c8shao@ucsd.edu (C.S.); hairili@ucsd.edu (H.L.); xdfu@ucsd.edu (X.-D.F.)

**Keywords:** alternative splicing, breast cancer, exon skipping, cancer metastasis, *DCUN1D5*

## Abstract

Aberrant alternative splicing (AS) is a hallmark of cancer and a potential target for novel anti-cancer therapeutics. Breast cancer-associated AS events are known to be linked to disease progression, metastasis, and survival of breast cancer patients. To identify altered AS programs occurring in metastatic breast cancer, we perform a global analysis of AS events by using RNA-mediated oligonucleotide annealing, selection, and ligation coupled with next-generation sequencing (RASL-seq). We demonstrate that, relative to low-metastatic, high-metastatic breast cancer cells show different AS choices in genes related to cancer progression. Supporting a global reshape of cancer-related splicing profiles in metastatic breast cancer we found an enrichment of RNA-binding motifs recognized by several splicing regulators, which have aberrant expression levels or activity during breast cancer progression, including SRSF1. Among SRSF1-regulated targets we found *DCUN1D5*, a gene for which skipping of exon 4 in its pre-mRNA introduces a premature termination codon (PTC), thus generating an unstable transcript degraded by nonsense-mediated mRNA decay (NMD). Significantly, distinct breast cancer subtypes show different *DCUN1D5* isoform ratios with metastatic breast cancer expressing the highest level of the NMD-insensitive *DCUN1D5* mRNA, thus showing high *DCUN1D5* expression levels, which are ultimately associated with poor overall and relapse-free survival in breast cancer patients. Collectively, our results reveal global AS features of metastatic breast tumors, which open new possibilities for the treatment of these aggressive tumor types.

## 1. Introduction

Following transcription, introns are removed from the pre-mRNA transcript and exons are joined to each other to produce the mature messenger RNA (mRNA), a process known as splicing [1]. The splicing reaction is carried out in the nucleus by the spliceosome, a dynamic and large ribonucleoprotein (RNP) complex composed of proteins and RNAs [1,2]. Different from constitutive splicing, where an exon is always included in the final mRNA, alternative splicing (AS) produces more than one mRNA isoform from a single pre-mRNA using various combinations of 5′ and 3′ splice-sites to achieve proteome diversity. In humans, at least 95% of pre-mRNAs undergo AS [3]. Different types of AS events arise from the different recognition of splice sites and include: Cassette exons or skipped exons (SE), alternative 5′ splice-site (A5SS), alternative 3′ splice-site (A3SS), alternative transcription start sites (AltS), alternative transcription termination sites (AltT), mutually exclusive exons (MEE), multi-exon skipping (MES), and intron retention (IR). These different types of AS are regulated by the concerted action of *cis*-acting sequences on the pre-mRNA target and *trans*-acting RNA-binding proteins (RBPs).

In addition to increasing protein diversity, AS can include premature termination codons (PTCs) to regulate mRNA stability [4,5], a process known as alternative splicing coupled to nonsense-mediated decay (AS-NMD). In mammals, a termination codon is considered premature and causes NMD when it is located ≥50–55 nucleotides (nt) upstream of an exon–exon junction bound by the exon junction complex (EJC) [6]. EJCs downstream of PTCs are no longer removed during the first “pioneer” round of translation and able to recruit important NMD factors such as UPF1, which promotes mRNA degradation [7].

Aberrant AS is frequently observed in cancer [8,9,10]. In particular, global analyses have shown that there are at least 15,000 cancer-specific AS variants from 27 types of cancers [11,12]. Importantly, a causative role of aberrant AS in tumor cells has also been provided [13,14]. Remarkably, the identification of cancer-related AS variants has supported the notion that splicing fidelity is lost during cancer progression [15,16,17], whereas a number of RBPs can act as bona fide oncoproteins [18]. Nevertheless, several splicing regulators act as tumor suppressors [19,20].

Breast cancer is one of the three most common cancers in women and the second leading cause of cancer deaths in women [21,22]. Notably, global transcriptome and mutational analyses have shown that a significant number of altered AS events occur in genes involved in breast cancer oncogenesis [11,12,23,24,25,26,27,28,29]. Furthermore, different RBPs are aberrantly expressed in breast tumors. Notably, more than 50% of human breast tumors have an alteration in at least one of SR family members [30], important factors for both constitutive and regulated splicing reactions.

RNA-mediated oligonucleotide annealing, selection, and ligation coupled with next-generation sequencing (RASL-seq) uses a pool of primer pairs that are specific to exon junctions to detect ~5530 known AS events conserved between human and mice [31]. As opposed to completely unbiased profiling of AS by RNA-seq, RASL-seq focuses on annotated targets without allowing de novo discovery of novel AS events. However, RASL-seq is robust in quantitatively determining expression differences of mRNA isoforms [31,32]. RASL-seq can be used to compare and characterize AS programs in different cells or patient samples [31,32,33]. Importantly, numerous annotated AS events related to cancer progression are included in RASL-seq categories.

Due to the relevance of AS dysregulation in cancer progression, we performed RASL-seq of MCF7 and MDA-MB-231 cells, representing low- and high-metastatic human breast cancer cell lines, respectively [34], to identify breast cancer metastasis-associated AS changes. Validation of RASL-seq result was then performed in different breast cancer cell lines, including MCF7, T47D, BT-549, and MDA-MB-231, thus demonstrating the effectiveness of RASL-seq in the identification of AS changes potentially involved in breast cancer progression.

Next, we investigated enrichment of RNA motifs located near or within the regulated AS cassette exons to discover candidate sequence-specific features regulating the identified AS changes.

Finally, supported by the relevance of *DCUN1D5* in different cancer types [35], we focused our attention on *DCUN1D5* exon 4, an AS change identified by our RASL-seq analysis.

Collectively, we demonstrated that there are significantly different regulated AS events in high-metastatic breast cancer cells compared to low-metastatic breast cancer cells. Among these is *DCUN1D5* exon 4, whose skipping generates a PTC-containing transcript degraded by the NMD pathway.

Accordingly, a decreased skipping of *DCUN1D5* exon 4 and, thus, an increase of total *DCUN1D5* expression in high-metastatic triple-negative breast cancer (TNBC) MDA-MB-231 cells were observed. Importantly, we also found a reduction of exon 4 skipping and high *DCUN1D5* expression in breast cancer specimens (vs. normal tissues) and in high-metastatic subtypes (vs. low-metastatic breast tumors). Mechanistically, we found that skipping of *DCUN1D5* exon 4 is regulated—at least in part—by SRSF1. Importantly, high expression levels of *DCUN1D5* were associated with a poor 5-year overall survival rate and relapse-free survival in breast cancer patients. Collectively, our data contribute to defining the impact of AS changes in breast cancer thus leading to a better comprehension of the molecular underpinnings involved in the malignant transformation.

## 2. Materials and Methods

### 2.1. Cell Culture, RNA Extraction, and RT-PCR

MCF7 [American Type Culture Collection (ATCC, HTB-22™)], MDA-MB-231 (ATCC, HTB-26™), T47D (ATCC, HTV-133) cells were cultured in RPMI medium (HyClone, Logan, UT, USA, Cat: SH30027.01) supplemented with 10% fetal bovine serum (FBS) (HyClone, Logan, UT, USA, Cat: SH30084.03), 2 mM glutamine (Gibco, Grand island, NY, USA, Cat: 35050-061), 1X penicillin-streptomycin (HyClone, Logan, UT, USA Cat: SV30010). BT549 (ATCC, HTB-122) cells were cultured in DMEM medium (HyClone, Logan, UT, USA, Cat: SH30243.01) supplemented with 10% fetal bovine serum (FBS) (HyClone, Logan, UT, USA, Cat: SH30084.03), 2 mM glutamine (Gibco, Grand island, NY, USA, Cat: 35050-061), 1X penicillin-streptomycin (HyClone, Logan, UT, USA Cat: SV30010). MCF10A (ATCC, CRL-10317) cells were cultured in DMEM/F12 medium (Invitrogen, San Francisco, CA, USA, Cat: 11330-032) supplemented with 5% Horse serum (Invitrogen, San Francisco, CA, USA, Cat: 16050-122), 20 ng/mL EGF (Peprotech, Rocky Hill, NJ, USA, Cat: AF-100-15), 0.5 mg Hydrocorisone (Sigma-Aldrich, St. Louis, MO, USA, Cat: H0888), 100 ng/mL Cholera toxin (Sigma-Aldrich, St. Louis, MO, USA, Cat: C8052), 10 ug/mL Insulin (Sigma-Aldrich, St. Louis, MO, USA, Cat: I1882), and 1X penicillin-streptomycin (HyClone, Logan, UT, USA, Cat: SV30010). Human embryonic kidney (HEK) 293 cells (ATCC, CRL-1573) and HeLa (ATCC, CCL-2) were cultured in high glucose (4.5 g/L) DMEM (Euroclone, Pero, Italy, Cat: ECM011L) supplemented with 10% FBS (Euroclone, Pero, Italy, Cat: ECS0180L), 4 mM glutamine (Euroclone, Pero, Italy, Cat: ECB3000D), and 100 U/L penicillin/streptomycin (Euroclone, Pero, Italy, Cat: ECB3001D). All cell lines were cultured at 37 °C in a 5% CO_2_ incubator.

HEK-293, HeLa, and MCF7 cells were treated with cycloheximide (CHX; 10 μg/mL; Sigma-Aldrich, St. Louis, MO, USA, Cat: 01810) or DMSO as control for 6 h.

Total RNAs were extracted using RixoEX reagent (GeneAll, Seoul, Korea, Cat: 301-001) or Rneasy Mini kit (Qiagen, Hilden, Germany, Cat: 74106) according to the manufacture’s instruction. Reverse transcription was performed using M-MLV reverse transcriptase (ELPIS, Daejeon, Korea, Cat: EBT-1028) and 1 μg RNA for cDNA synthesis. Then, 0.5 μL cDNA was used for PCR amplification. Quantitative RT-PCR (RT-qPCR) was performed with iQ™ SYBR^®^ Green Supermix kit (BioRad, Hercules, CA, USA, Cat: 1708880) or QuantiTect SYBR Green PCR (Qiagen, Hilden, Germany, Cat: 204145) using a LyghtCycler 480 (Roche, Basel, Switzerland), according to the manufacturer’s instructions using GAPDH, B2M, or RPLP0 as an internal control. Primers used in this study are listed in Supplementary Table S1.

### 2.2. Alternative Splicing Analysis with RASL-Seq

A pool of oligonucleotides was designed to detect 5530 AS events. RASL reaction was performed as previously described. Two oligonucleotide sets were designed to detect mRNA isoforms of one gene with cassette exon included and excluded. The mixture of oligonucleotides was hybridized with RNAs and selected with biotin-labeled oligo dT. Two nearby oligos were then ligated and barcoded for high-throughput sequencing using Illumina Hiseq 2500 apparatus (Illumina, San Diego, CA, USA). Splicing events were filtered for a minimum of 5 read counts in all biologic triplicates. AS changes were filtered using the following criteria: Ratio change of at least 2 and *p*-value < 0.05. Gene enriched in up-, down- and non-differentially regulated (ndiff) AS events in high-metastatic breast cancer cells are listed in Supplementary Table S2.

### 2.3. Gene Ontology (GO) Analysis

GO analysis was performed using DAVID Bioinformatics Resources 6.8 (https://david.ncifcrf.gov/; accessed on 10 July 2020) [36].

### 2.4. Calculation of Splicing Score

3′ splice site sequences including 20 nt of 3′ end intron and 3 nt of 5′ exon were used to analyze 3′ splice-site score using MaxEntScan [37,38]. 9 nt sequences containing 3 nt of 3′ exon and 6 nt of 5′ intron were used to calculate 5′ splice-site scores using MaxEntScan. Cassette exon length, intron length, median transcript length, and GC content were calculated after extracting sequences using BEDTools [37]. RNA-binding motifs were analyzed using Discriminative Regular Expression Motif Elicitation (DREME) algorithm [39].

### 2.5. RNA-Binding Motifs Prediction

SRSF1 RNA-binding motifs predictions for selected genes were also performed by using RBPmap web server (http://rbpmap.technion.ac.il; accessed on 3 March 2021) [40], with the following parameters: Stringency level = low, SpliceAid 2 (http://www.introni.it/spliceaid.html; accessed on 3 March 2021) [41], and ESEfinder, only for SRSF1 (http://krainer01.cshl.edu/cgi-bin/tools/ESE3/esefinder.cgi?process=home; accessed on 3 March 2021) [42,43].

### 2.6. Gene Expression and Splicing Analyses of Transcriptomic Data

Expression analysis of RBPs, whose binding motifs were enriched in up and down identified cassette exons, in breast specimens (normal breast; luminal breast cancer; HER2 positive breast cancer; TNBC) of the TCGA-BRCA dataset was performed by using UALCAN web-tool (http://ualcan.path.uab.edu; accessed on 3 March 2021) [44].

*DCUN1D5* mRNA splicing patterns (skipping of exon 4) were analyzed using TCGA SpliceSeq (http://projects.insilico.us.com/TCGASpliceSeq; accessed on 3 March 2021) [45], a web-based resource known to provide Percent Splice-In (PSI) values for splicing events retrieved in specimens from the TCGA-BRCA level 3 dataset. TCGA-BRCA cancer omics data were also analyzed using UALCAN web-tool (http://ualcan.path.uab.edu; accessed on 3 March 2021) to determine total *DCUN1D5* expression levels. Transcripts per million (TPM) data were used to generate boxplots. Interquartile ranges (minimum, 25th percentile, median, 75th percentile, maximum) are shown. UALCAN also provides an estimate of statistically significant differences in gene expression level between groups employing TPM values and a student’s *t*-test. Gene expression data were also retrieved using the Oncomine tool (http://oncomine.org/resource; accessed on 3 March 2021) [46]. Breast cancer microarray databases with altered *DCUN1D5* gene expression were filtered considering their significance (*p*-value < 0.05). Log_2_-median centered intensity is shown. Additional breast cancer datasets (Perou GSE3521 and GSE10893) were retrieved using the Human Cancer Metastasis Database (HCMDB) (https://hcmdb.i-sanger.com; accessed on 3 March 2021) [47]. Gene expression data are shown as Log_2_ intensity. HCMDB also provides breast transcriptome data classified based on metastasis status.

Gene and transcript expression levels in breast cancer cell lines were retrieved in the Cancer Cell Line Encyclopedia database (CCLE; https://portals.broadinstitute.org/ccle/; accessed on 3 March 2021) [48]. Breast cancer cell lines were classified as luminal, HER2 positive, or TNBC according to Dai et al., 2017 [49]. Percentage of cassette exon inclusion of each analyzed event in the CCLE was calculated considering all the annotated transcripts in which the cassette exon is present of total transcripts in which the alternative region is transcribed (at least one constitutive exon should be present upstream and downstream the alternative exon).

### 2.7. Survival Analysis

Overall survival curves of breast cancer patients were generated by analyzing clinical data (survival time and survival status) of patients with breast cancer of the TCGA-BRCA dataset. Patients were stratified (split by median) according to *DCUN1D5* RSEM expression. Clinical data and RNA-seq data of *DCUN1D5* expression were obtained from TSVdb web-tool (http://www.tsvdb.com; accessed on 3 March 2021) [50]. Log-rank Mantel–Cox test was employed to determine any statistical difference between the survival curves. Additional 5-years relapse-free survival curves were obtained from a Kaplan–Meier Plotter (https://kmplot.com/analysis; accessed on 3 March 2021) [51] with indicated probes for *DCUN1D5*. Hazard ratio (and 95% confidence intervals) and log-rank P are displayed.

### 2.8. Plasmids

T7-tagged SRSF1 vector was generated as described previously [52]. SRSF3-T7 plasmid was previously described [52]. *DCUN1D5*-GFP expression plasmid was produced by PCR-amplification using human genomic DNA as a template and inserting in pEGFP-C1 vector with HindIII/BamHI enzymes. Primers used are listed in Supplementary Table S1.

### 2.9. Plasmid Transfection and siRNA Silencing

MCF7 and HEK-293 cells were transiently transfected with lipofectamine LTX with Plus reagent (Invitrogen, Cat: 15338100), according to the manufacturer’s protocol. Briefly, cells were seeded in a 12-well or 96-well plate in order to reach 70–90% of confluence the day of the transfection. SRSF1 silencing in MCF7 cells was performed by using three consecutive RNAiMax Lipofectamine (Life Technologies, Waltham, MA, USA, Cat: 13778075) transfection of 30 pmol of SRSF1 siRNA (gcaacagcaggagucgcaguu/cugcgacuccugcuguugcuu) or a control siRNA.

### 2.10. Immunoblotting

Transfected MCF7 and HEK-293 cells were lysed in Laemmli buffer, supplemented with protease and phosphatase inhibitors (cOmplete™ and EDTA-free Protease Inhibitor cocktail; Roche, Basel, Switzerland, Cat: 1187350001). Proteins were separated in SDS–PAGE and analyzed by Western blotting by using standard procedures. Membranes were blocked by incubation with 5% non-fat dry milk and the following primary antibodies were used: Anti-GFP (1:1000; Millipore, Burlington, MA, USA, Cat: MAB3580); anti-T7 (1:10,000; Millipore, Burlington, MA, USA, Cat: 69522-3); anti-Vinculin (1:5000; Millipore, Burlington, MA, USA, Cat: MAB3574). The following secondary antibody linked to horseradish peroxidase was used: Anti-Mouse (1:5000; West Grove, Jackson ImmunoResearch, PA, USA, Cat: 115-035-146). Immunostained bands were detected using the chemiluminescent method (LiteAblot Plus; Euroclone, Pero, Italy, Cat: EMP011005).

### 2.11. Cell Imaging

*DCUN1D5* transiently transfected MCF7 cells were fixed after 24 h with 4% paraformaldehyde (PFA; Sigma-Aldrich, St. Louis, MO, USA, Cat: P6148). Nuclei were stained with 0.1 g/mL DAPI (Sigma-Aldrich, St. Louis, MO, USA, Cat: MBD0015). For imaging, an epifluorescence microscope (Optical Microscope Olympus IX71) equipped with a 60X objective was used. Photomicrographs were taken with a digital camera Cool SNAPES (Photometrics, Tucson, AZ, USA). Data acquisition was done using the MetaMorph 7.7.5 software (Universal Imaging Corporation, Bedford Hills, NY, USA).

### 2.12. Cell Proliferation Assay

MCF7 proliferation of untransfected, EGFP-C1 transfected, and *DCUN1D5*-GFP transfected cells was evaluated after 24 h upon transfection in a 96-well plate by fixing cells with 4% paraformaldehyde (PFA; Sigma-Aldrich, St. Louis, MO, USA, Cat: P6148) and staining it with crystal violet (Gibco, Grand Island, NY, USA, Cat: 42555) solution (0.1% in 20% methanol). For quantification, stained crystal violet was solubilized with 10% acetic acid. Absorbance was read at 620 nm (two measurements) with an EZ Read 400 microplate reader (Biochrom, Cambridge, UK).

### 2.13. Statistical Analyses and Plotting

Two-sided *t*-tests were performed for all splice-site strength analyses [38]. Ordinary one-way ANOVA for multiple comparisons was performed to compare more than 2 groups. Mann–Whitney two-sided U test was performed for transcript median length, exon length, upstream intron length, and GC content [53]. Plots in Figure 3 are plotted with R (http://www.R-project.org/; accessed on 25 February 2021).

## 3. Results

### 3.1. Identification of Widespread AS Changes in High-Metastatic Breast Cancer Cells

**Figure 1:**
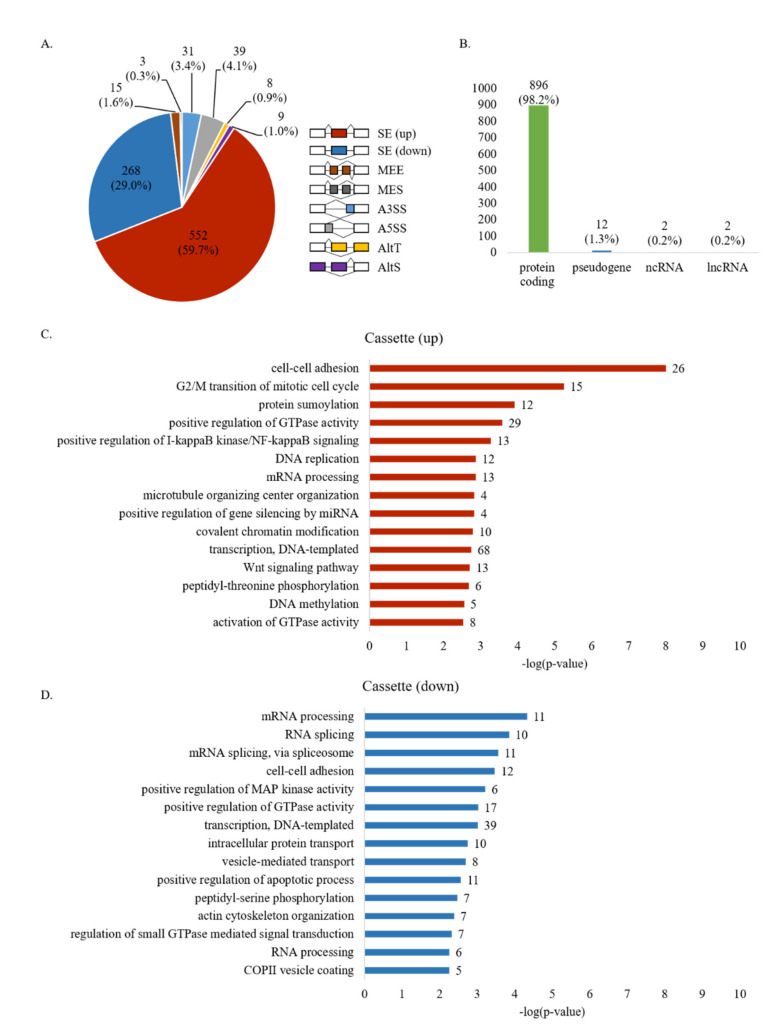
Pre-mRNAs undergo global aberrant AS in high-metastatic breast cancer cells. (**A**) Pie chart showing various AS events from RNA-mediated oligonucleotide annealing, selection, and ligation coupled with next-generation sequencing (RASL-seq) results. All events listed are drawn in the right. Increased exon inclusion is indicated as “up”, and increased exon skipping is indicated as “down”, (**B**) Gene distribution of RASL-seq results. (**C**) Gene ontology (GO) analysis of genes enriched in up-regulated AS cassette exons in high-metastatic breast cancer cells. (**D**) GO analysis of genes enriched in down-regulated AS cassette exons in high-metastatic breast cancer cells.

To identify pre-mRNAs undergoing abnormal AS regulation in high-metastatic breast cancer cells compared to low-metastatic cancer cells, we performed a RASL-seq using RNAs extracted from MDA-MB-231 and MCF7 cell lines, respectively. RASL oligonucleotide pool was designed to detect 5530 AS events in the human genome. Collectively, we detected 1558 AS events that expressed both isoforms in MCF7 and MDA-MB-231 cells with a minimum of 5 read counts for each isoform, allowing calculating isoform ratio changes. By analyzing biological triplicates, AS switches of these two cell lines with ratio changes ≥2 and ≤−2 were considered to be significantly (*p* < 0.05) up-regulated and down-regulated splicing events, respectively. Conversely, AS events with the ratio change comprised between 2 and −2 were considered to be non-differentially regulated (ndiff). Using this cutoff, we identified 925 AS events—~59.4% of a total of 1558 identified—significantly altered in MDA-MB-231 cells compared to MCF7 cells, suggesting a striking difference in splicing programs between these two cell lines (Supplementary Table S2). In MDA-MB-231 cells, 552 and 268 SEs exhibited significantly increased or decreased ratios compared to those in MCF7 cells (Figure 1A). RASL-seq mostly represents AS of SEs, although it can also be used to investigate other types of AS events. Accordingly, we were also able to detect 39 A5SS, 31 A3SS, 15 MEE, 3 MES, 9 AltS, and 8 AltT (Figure 1A) (Supplementary Table S2). Importantly, most of the regulated AS events (98.2%) affected protein-coding genes, further supporting the importance of AS in the generation of human protein diversity (Figure 1B). Taken together, RASL-seq results reveal global AS switches associated with the metastatic potential of MDA-MB-231 cells.

To identify the functional impact of the altered AS events in metastatic cancer cells, we next applied Gene Ontology (GO) analysis to locate functional categories enriched in the set of genes showing increased or decreased cassette exons splicing. As shown in Figure 1C,D, among the mainly enriched terms are biological processes frequently altered during tumor progression and metastasis spread. In particular, GO terms for up-regulated cassette exon splicing included cell–cell adhesion (GO:0098609), G2/M transition of mitotic cell cycle (GO:0000086), DNA replication (GO: 0006260), mRNA processing (GO:0006397), and Wnt signaling (GO:0016055) (Figure 1C, Supplementary Table S3), whereas biological processes of genes with down-regulated cassette exon splicing showed enrichment for genes involved in mRNA processing (GO:0006397), cell–cell adhesion (GO:0098609), positive regulation of GTPase activity (GO:0043547), and transcription (GO:0006351) (Figure 1D, Supplementary Table S3). The explanations of each category are listed in Supplementary Table S3.

### 3.2. Validation of RASL-Seq Results in High- and Low-Metastatic Breast Cancer Cells

**Figure 2:**
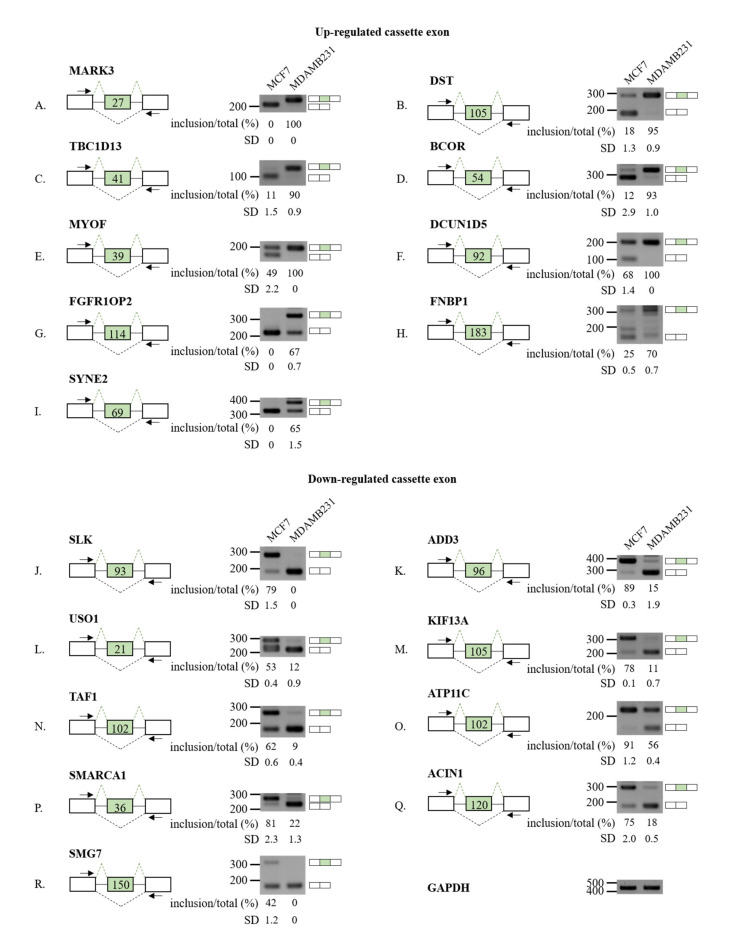
Validation of AS cassette exons up (**A**–**I**) or down (**J**–**R**) regulated in high-metastatic vs. low-metastatic breast cancer cells. (**A**–**R**) (Left) For each gene, the schematic representation of the genomic region containing the AS cassette exon is shown. Lengths of cassette exons are shown in the green box. Inclusions of cassette exons are shown with dotted green lines. Skipping events are shown with dotted black lines. Primers used in RT-PCR are shown with arrows. (Right) RT-PCR analysis (in triplicated) of the AS profile of cassette exons was performed by using RNA extracted from MCF7 and MDA-MD-231. Quantitation results are shown at the bottom of each gel. Standard deviations (SD) calculated from three independent experiments are shown.

To validate our RASL-seq results, we performed RT-PCR analysis of 18 selected AS cassette exons (9 from up-regulated and 9 from down-regulated ones) using RNAs extracted from high-metastatic MDA-MD-231 cells or low-metastatic MCF7 cells. Significant increases of exon inclusion were confirmed for *microtubule affinity regulating kinase 3* (*MARK3*) exon 15 (Figure 2A), *dystonin* (*DST*) exon 93 (Figure 2B), *TBC1 domain family member 13* (*TBC1D13*) exon 3 (Figure 2C), *BCL6 corepressor* (*BCOR*) exon 4 (Figure 2D), *myoferlin* (*MYOF*) exon 17 (Figure 2E), *defective in cullin neddylation 1 domain containing 5* (*DCUN1D5*) exon 4 (Figure 2F), *FGFR1 oncogene partner 2* (*FGFR1OP2*) exon 4 (Figure 2G), *formin binding protein 1* (*FNBP1*) exon 10 (Figure 2H), and *spectrin repeat containing nuclear envelope protein 2* (*SYNE2*) exon 106 (Figure 2I). Conversely, decrease of exon inclusion was validated for *STE20 like kinase* (*SLK*) exon 13 (Figure 2J), *adducin 3* (*ADD3*) exon 13 (Figure 2K), *USO1 vesicle transport factor* (*USO1*) exon 15 (Figure 2L), *kinesin family member 13A* (*KIF13A*) exon 38 (Figure 2M), *TATA-box binding protein associated factor 1* (*TAF1*) exon 36 (Figure 2N), *ATPase phospholipid transporting 11C* (*ATP11C*) exon 29 (Figure 2O), *SWI/SNF related*, *matrix associated*, *actin dependent regulator of chromatin*, *subfamily a*, *member 1* (*SMARCA1*) exon 13 (Figure 2P), *apoptotic chromatin condensation inducer 1* (*ACIN1*) exon 4 (Figure 2Q), and *SMG7 nonsense mediated mRNA decay factor* (*SMG7*) exon 18 (Figure 2R). Our results demonstrate that RASL-seq is a highly efficient and robust tool for identifying global AS changes.

To further prove that our RASL-seq in MCF7/MDA-MD-231 was also effective in the identification of differently regulated AS events in highly metastatic breast cancer cells, we have also validated the 18 cassette exons shown in Figure 2 in another model of low-metastatic versus high-metastatic breast cancer, namely T47D and BT-549 cells. As shown in the Supplementary Figure S1, we found that 16 out of 18 events (*MARK3* exon 15; *DST* exon 93; *TBC1D13* exon 3; *MYOF* exon 17; *DCUN1D5* exon 4; *FGFR1OP2* exon 4; *FNBP1* exon 10; *SYNE2* exon 106; *SLK* exon 13; *ADD3* exon 13; *USO1* exon 15; *KIF13A* exon 38; *ATP11C* exon 29; *SMARCA1* exon 13; *ACIN1* exon 4; *SMG7* exon 18) were in the same direction (inclusion or skipping) also in low-metastatic T47D compared to high-metastatic BT-549 breast cancer cell lines. Moreover, we also exploited the CCLE [48] database to analyze inclusion levels of the aberrantly regulated cassette exons in a total of 46 breast cancer cell lines classified according to their major subtype (luminal; HER2 positive, or TNBC). Notably, a significant increase in exon inclusion in high-metastatic TNBC was found for *MARK3*, *MYOF*, *FGFR1OP2*, and *FNBP1* events. Conversely, increased skipping of cassette exons in TNBC cells was observed for *SLK*, *ADD3*, *USO1*, and *KIF13A* (Supplementary Figure S2 and Supplementary Table S4). Collectively, our results showed a global splicing modification of metastatic breast cancer cells.

### 3.3. Distinct Sequence Properties of AS Cassette Exons Differently Regulated in High- vs. Low-Metastatic Breast Cancer Cells

**Figure 3:**
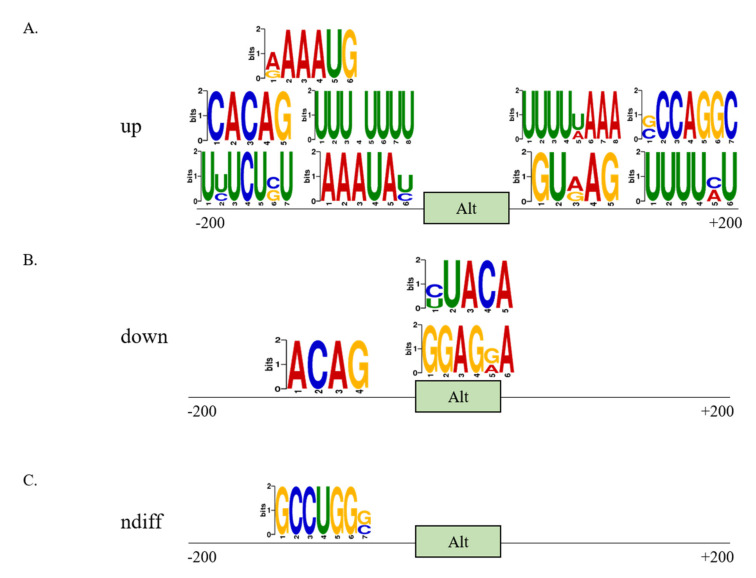
RNA sequences motifs enriched in up- (**A**), down- (**B**) regulated AS cassette exons, and in non-differentially regulated exons (**C**). Green boxes = alternative cassette exons.

As widespread differences of AS events were observed in high-metastatic MDA-MB-231 cells, we wanted to understand sequence features of enhanced or repressed AS cassette exons in these metastatic cells. The outcome of AS reaction depends on the coordinated action of *trans*-acting RBPs that bind *cis*-regulatory sequences within the nascent pre-mRNA and promote inclusion or skipping of specific AS exons. Thus, we first looked for enrichment of RNA motifs associated with the metastasis potential of MDA-MB-231 cells across AS cassette exons and their flanking intronic sequences (200 nt). In up-regulated cassette exons, we found that numerous motifs were enriched in upstream (RAAAUG, CACAG, UUUNUUUU, UYUCUSU, AAAUAY) and downstream (UUUUWAAA, SCCAGGC, GURAG, UUUUMU) introns, but not inside the AS exons (Figure 3A). Additionally, in down-regulated cassette exons, we found enriched motifs located in the regulated exons (YUACA, GGAGRA) or in the upstream intron (ACAG) (Figure 3B). In ndiff cassette exons, we could detect only a motif (GCCUGGS) located at upstream intron (Figure 3C). RBPs that might bind to these motifs are listed in Supplementary Table S5.

Importantly, we found that several RBPs, which are able to recognize the enriched sequences at 5′ and 3′ splice-sites and inside the AS cassette exons, were differentially expressed in breast cancer and during breast tumor progression, including PTBP1 [54,55], SRSF1 [28,30], and SRSF9 [30].

We further compared splice site strength of up-regulated (n = 552) and down-regulated (n = 268) cassette exons in MDA-MB-231 cells vs. MCF7 cells as well as non-differentially regulated exons (ndiff) (n = 307). To this aim, we compared 5′ and 3′ splice-site scores of cassette exon (Alt) and its flanking constitutive exons (C1 and C2) by obtaining splice-site scores of each of the four splice-sites (Supplementary Figure S3A). As shown in Supplementary Figure S3B, flanking exons included similar strength of 5′ and 3′ splice-sites. In addition, we were not able to observe differences in 3′ splice-site strength of cassette exons (in either up- and down-regulated exons) and 5′ splice-site strength in the down-regulated exons (Supplementary Figure S3B). However, we found that up-regulated cassette exons have a stronger 5′ splice-site than non-differentially regulated exons (Supplementary Figure S3B).

It has been also shown that intron–exon architecture affects splicing site recognition. For example, short introns favor the “intron definition” model for exon skipping decisions, whereas long introns favor the “exon definition” and exon skipping in some genes [56]. Additionally, differential exon–intron GC content also regulates inclusion levels of alternatively spliced exons by affecting nucleosome occupancy and recruitment of splicing factors on the nascent pre-mRNA [57]. Thus, we analyzed the length of introns and exons as well as GC contents of flanking introns of cassette exons. As shown in Supplementary Figure S3C, up-regulated exons had shorter exon length and lower GC contents. We also observed that down-regulated cassette exons contained shorter upstream intron and higher GC contents, but not the differences in exon length (Supplementary Figure S3C). Collectively, these pre-mRNA intrinsic features could contribute to splice site selection and AS determination.

### 3.4. DCUN1D5 Expression Levels Are Significantly Associated with Breast Cancer Metastasis, and Survival

**Figure 4:**
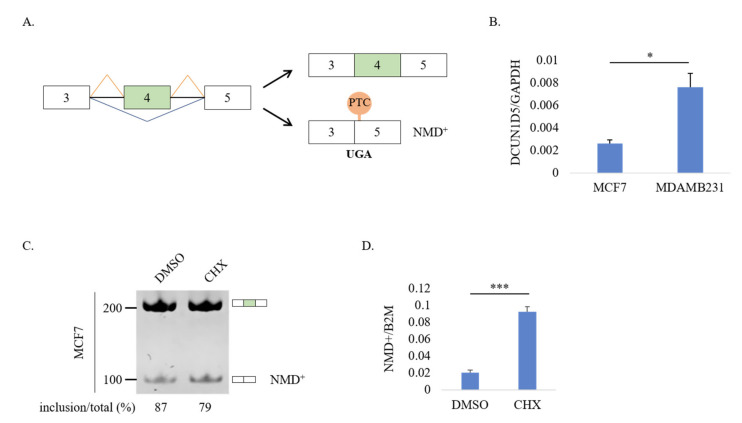
The alternatively spliced mRNA of *DCUN1D5* deleted of exon 4 is degraded by NMD pathway. (**A**) Schematic representation of the AS regulation of *DCUN1D5* exon 4. Skipping of exon 4 (blue lines) results in the introduction of a premature stop-codon (PTC, orange balloon) in exon 5, thus leading to the generation of an NMD sensible transcript (NMD+). (**B**) *DCUN1D5* mRNA expression levels by RT-qPCR in MCF7 and MDA-MB-231 cells. (**C**) RT-PCR analysis of the AS profile of *DCUN1D5* exon 4 in MCF7, treated with DMSO or cycloheximide (CHX). CHX is able to stabilize the NMD+ transcript of *DCUN1D5*. (**D**) RT-qPCR analysis of NMD+ *DCUN1D5* mRNA. Student *t*-test. Error bars indicate standard deviation (SD) calculated from three independent experiments, *** *p* < 0.001, * *p* < 0.05.

Among the validated AS events (Figure 2), we focused on exon 4 (ENSE00003623357) of Defect in cullin neddylation 1 domain containing 5 (*DCUN1D5*, a.k.a., squamous cell carcinoma related oncogene 5, SCCRO5), a member of the DCUN protein family characterized by the presence of a conserved C-terminal potentiating of neddylation (PONY) domain required for neddylation, a post-translational modification interesting a complex network of protein substrates [58,59,60]. *DCUN1D5* was found to be up-regulated in different cancer types, whereas its elevated expression levels were reported to increase cell proliferation, migration, and invasion [35], thus suggesting the involvement of *DCUN1D5* in cancer progression. *DCUN1D5* exon 4 was preferentially included in high-metastatic MDA-MB-231 cells compared to low-metastatic MCF7 (Figure 2F). As shown in Figure 4A, skipping of *DCUN1D5* exon 4 introduces in the mature mRNA a PTC that could generate an unstable transcript (that we called NMD+ *DCUN1D5)* degraded through the NMD pathway. Thus, the coupling of AS of *DCUN1D5* exon 4 and NMD (AS-NMD) could be an important mechanism that controls the total *DCUN1D5* expression levels in the cell. In line with this hypothesis, Tani and colleagues [61] found that *DCUN1D5* mRNA is one of the targets of UPF1, a key NMD player [62]. In support of the AS-NMD involvement, we found that the inclusion of *DCUN1D5* exon 4 was accompanied by higher *DCUN1D5* total mRNA expression in MDA-MB-231 cells compared to MCF7 cells (Figure 4B). In addition, we also found that *DCUN1D5* splicing profiles parallel *DCUN1D5* expression levels in a wide panel of breast cancer cell lines present in the Cancer Cell Line Encyclopedia (CCLE) database (Supplementary Figure S4). In particular, breast cancer cell lines with high metastatic potential (TNBC subtype) showed high levels of *DCUN1D5* exon 4 inclusion and high *DCUN1D5* expression levels compared to breast cancer cell lines with low metastatic potential (luminal subtype) (Supplementary Figure S4A,B). Based on these results, we next wondered whether the AS-NMD might be involved in regulating *DCUN1D5* expression in metastatic breast cancer cells. To demonstrate the involvement of the NMD pathway in regulating *DCUN1D5* expression, we treated MCF7 breast cancer cells with cycloheximide (CHX), a well-known NMD inhibitor [63]. As shown in Figure 4C,D, CHX significantly stabilized the expression of the NMD+ *DCUN1D5* transcript as detected by RT-PCR and RT-qPCR (Figure 4C,D). These results were also confirmed in two other cell lines (HEK-293 and HeLa) known to express *DCUN1D5* (Supplementary Figure S5A,D). Notably, we also found a significant negative correlation (r = −0.35; *p*-value = 0.016) between the percentage of NMD+ transcripts and total *DCUN1D5* expression in breast cancer cell lines of the CCLE database (Supplementary Figure S4C).

To support the involvement of *DCUN1D5* in breast cancer cell biology, we overexpressed GFP-tagged *DCUN1D5* in low-metastatic MCF7 cells. As shown in Supplementary Figure S6, we confirmed that recombinant *DCUN1D5*-GFP localized in the nucleus (as previously reported) where it is required for its oncogenic function [58] (Supplementary Figure S6A,B). Importantly, we found that overexpression of *DCUN1D5* increased the proliferation of MCF7 cells (Supplementary Figure S6C).

**Figure 5:**
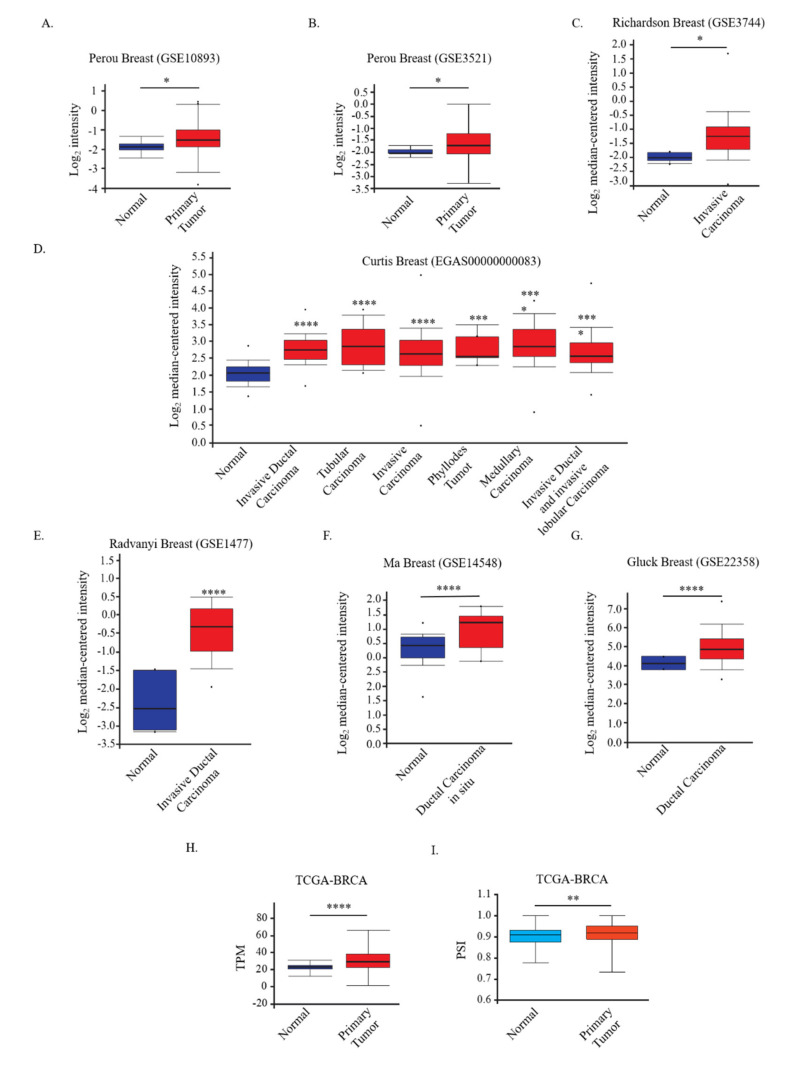
*DCUN1D5* is highly expressed and aberrantly spliced in breast tumors. (**A**–**G**) Microarray analyses of *DCUN1D5* mRNA expression in specimens of Perou Breast (GSE10893) (**A**), Perou Breast (GSE3521) (**B**), Richardson Breast (GSE3744) (**C**), Curtis Breast (EGAS00000000083) (**D**), Radvanyi Breast (GSE1477) (**E**), Ma Breast (GSE14548) (**F**), and Gluck Breast (GSE22358) (**G**) datasets. (**H**,**I**) Total *DCUN1D5* mRNA expression levels [shown with transcript per million (TPM)] (**H**) and *DCUN1D5* exon 4 splicing [PSI= Percent Splice-inclusion] (**I**) in normal and primary tumor specimens from the TCGA-BRCA dataset. Box blots indicate *DCUN1D5* expression as Log_2_-median centered intensity, Log_2_ Intensity, or TPM as indicated. **** *p* < 0.0001, *** *p* < 0.001, ** *p* < 0.01, * *p* < 0.05.

To determine the *DCUN1D5* expression levels in breast tumors, we interrogated various cancer databases. We found that *DCUN1D5* expression was significantly higher in primary tumor samples than in normal samples in Perou breast cancer datasets (GSE10893 and GSE3521) (Figure 5A,B). Moreover, we also found higher *DCUN1D5* expression in different types of breast tumors (invasive carcinoma, invasive ductal carcinoma, tubular carcinoma, phyllodes tumor, ductal carcinoma in situ, and medullary carcinoma) retrieved from microarray datasets [Richardson Breast (GSE3744), Curtis Breast (EGAS00000000083), Radvanyi Breast (GSE1477), MA Breast (GSE14548), and Gluck Breast (GSE22358)] compared to normal breast tissues (http://oncomine.org/resource/; accessed on 3 March 2021) (Figure 5C–G) [46].

To further investigate *DCUN1D5* expression in breast cancer, we used The Cancer Genome Atlas (TCGA-BRCA) [64] to compare non-pathological samples with primary breast tumors. As shown in Figure 5H, we confirmed *DCUN1D5* up-regulation in tumor specimens compared to normal ones using UALCAN web-tool (http://ualcan.path.uab.edu; accessed on 3 March 2021) [44]. RNA-seq TCGA dataset also allows the quantification of AS events that can be retrieved by using the TCGA Spliceseq web-tool (https://bioinformatics.mdanderson.org/TCGASpliceSeq; accessed on 3 March 2021) [45]. Importantly, we found a reduction of *DCUN1D5* exon 4 skipping, which sustains the production of the *DCUN1D5* NMD+ transcript, in tumors compared to normal breast tissues (Figure 5I).

**Figure 6:**
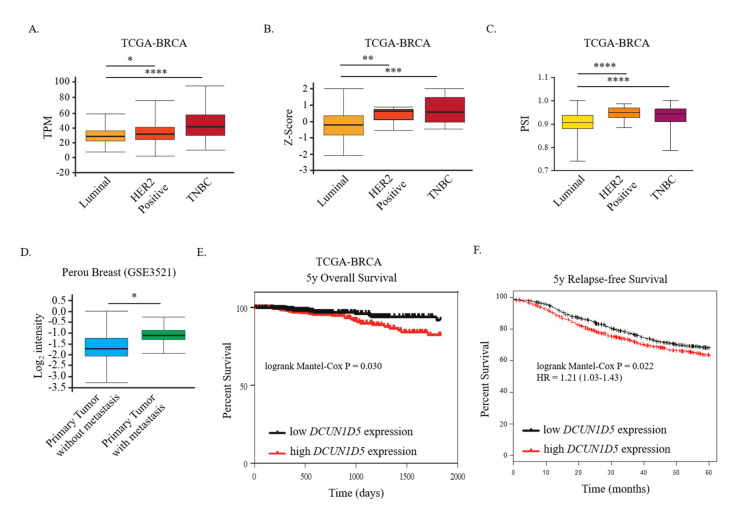
Increased *DCUN1D5* expression is characteristic of metastatic breast tumors and associated with low 5-year survival rate and relapse-free survival of breast cancer patients. (**A**) Total *DCUN1D5* mRNA expression, (**B**) *DCUN1D5* protein levels, and (**C**) *DCUN1D5* exon 4 splicing (PSI) in breast tumors of TCGA-BRCA dataset classified according to their subclasses (luminal; HER2 positive; TNBC). (**D**) Microarray analyses of *DCUN1D5* expression in breast tumor with or without distant metastasis of the Perou Breast (GSE3521) dataset. Box plots indicate *DCUN1D5* expression as Log_2_ intensity. (**E**) 5-year overall survival rate comparison of patients (TCGA-BRCA dataset) with higher (red) and lower (black) *DCUN1D5* expression (split by median). **** *p* < 0.0001, *** *p* < 0.001, ** *p* < 0.01, * *p* < 0.05. (**F**) 5-year relapse-free survival rate comparison of patients with higher (red) or lower (black) *DCUN1D5* expression (split by median) obtained from Kaplan–Maier Plotter. log-rank *p*-value and Hazard risk (HR) are indicated.

We next wondered whether *DCUN1D5* was expressed differently in metastatic breast tumors compared to non-metastatic tumors. Strikingly, as showed in Figure 6A, we found high *DCUN1D5* mRNA expression levels in high-metastatic triple-negative (TNBC) and HER2 positive breast cancers compared to low-metastatic luminal tumors annotated in the TCGA-BRCA dataset. Notably, protein expression of *DCUN1D5* was also up-regulated in both TNBC and HER2 positive breast tumors compared to luminal ones as determined using data from the Clinical Proteomic Tumor Consortium (CPTAC) Confirmatory/Discovery dataset retrieved by UALCAN web-tool (Figure 6B). Furthermore, *DCUN1D5* exon 4 skipping is highly reduced in highly metastatic TNBC and HER2 positive breast tumors than luminal breast tumors (Figure 6C). Accordingly, we found high *DCUN1D5* expression in breast cancer tissues with metastasis at distant sites compared to breast tumor without metastasis [Perou breast cancer dataset (GSE3521)] (Figure 6D). Collectively, these results indicate that *DCUN1D5* exon 4 skipping and as a consequence, the expression of the NMD+ *DCUN1D5* transcript inversely correlates with total (mRNA and protein) *DCUN1D5* levels in breast cancer specimens. Remarkable, low *DCUN1D5* exon 4 skipping and high *DCUN1D5* expression levels are found in highly metastatic breast tumors such as TNBC and HER2 positive breast tumors. Nevertheless, even if we could not rule out the existence of additional mechanisms regulating total *DCUN1D5* expression levels, our data suggest that an AS-NMD program activated by *DCUN1D5* exon 4 skipping could contribute, at least in part, to control *DCUN1D5* expression in breast cancer cells.

Finally, we compared 5-year overall survival rate of breast cancer patients with high and low *DCUN1D5* expression levels. As shown in Figure 6E, patients who expressed high *DCUN1D5* levels in breast tumors from the TCGA-BRCA dataset exhibited a significantly lower survival rate than patients with lower *DCUN1D5* expression. Notably, 5-year relapse-free survival rate was also decreased in breast cancer patients with high *DCUN1D5* expression retrieved from Kaplan–Maier Plotter (https://kmplot.com/analysis/; accessed on 3 March 2021) [51] (Figure 6F).

Collectively, our results strongly support a relevant role of high *DCUN1D5* expression levels and reduced exon 4 skipping during metastatic progression of breast cancer.

### 3.5. Molecular Mechanisms Regulation DCUN1D5 Exon 4 Splicing

**Figure 7:**
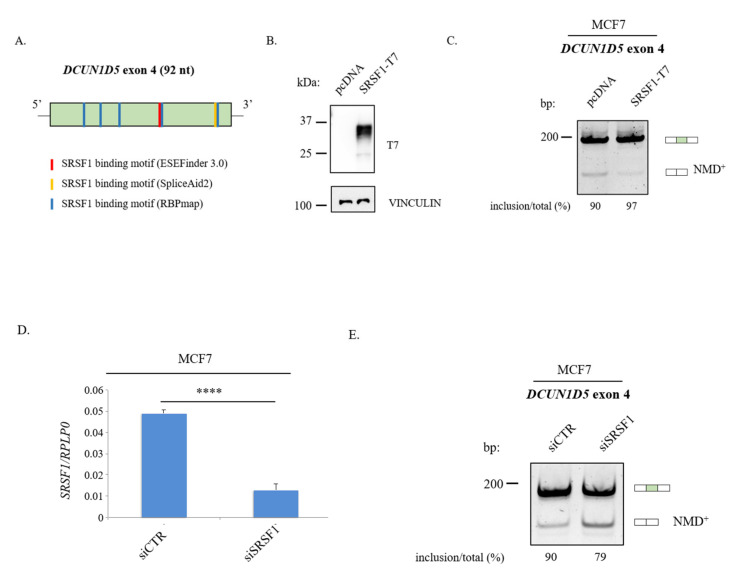
SRSF1 promotes *DCUN1D5* exon 4 inclusion. (**A**) Putative SRSF1 binding motifs are present in *DCUN1D5* exon 4. Red bars ESEFinder 3.0 prediction, yellow bars SpliceAid2 prediction, and blue bars RBPmap prediction (high stringency). (**B**) MCF7 cells were transfected with the T7-tagged SRSF1 or the empty (pcDNA3.1) vector. Expression of T7-SRSF1 was verified by immunoblotting with an anti-T7 antibody (α-VINCULIN as loading control). (**C**) RT-PCR analysis of *DCUN1D5* exon 4 splicing in the same transfected cells. (**D**) *SRSF1* mRNA expression levels by RT-qPCR in *SRSF1*-depleted MCF7 cells. (**E**) RT-PCR analysis of *DCUN1D5* exon 4 splicing upon SRSF1 depletion in breast MCF cells. Quantification of exon inclusion is shown below the gel. Student *t*-test. Error bars indicate standard deviation (SD) calculated from three independent experiments. **** *p* < 0.0001.

In order to decipher the molecular mechanism regulating *DCUN1D5* splicing, we analyzed the sequence of exon 4 to identify splicing enhancers (Exonic Splicing Enhancer or ESE) that could represent the binding sites for RBPs showed in Supplementary Table S5. By using ESEFinder 3.0 web tool [43], we were able to identify a putative ESE responsive to the human SRSF1 (Figure 7A; Supplementary Table S6), a member of the SR family of splicing regulators, which is frequently upregulated in different cancers [65]. Importantly, putative SRSF1 binding motifs were also predicted by using two additional tools such as RBPmap [40] and SpliceAid2 [41] (Figure 7A; Supplementary Table S6). To test the hypothesis that SRSF1 could regulate *DCUN1D5* exon 4 splicing we have transiently transfected MCF7 cells with a plasmid overexpressing the T7-tagged SRSF1 protein or depleted SRSF1 by using a siRNA-mediated knockdown approach. As shown in Figure 7B,C, we found that SRSF1 overexpression was able to promote *DCUN1D5* exon 4 inclusion in MCF7 cells, whereas its depletion increases the production of the *NMD+* transcript (Figure 7D,E). Similarly, this result was also confirmed in another cell line (HEK-293) (Supplementary Figure S7A,B). Notably, overexpression of different SR proteins, such as SRSF3, was not able to alter the *DCUN1D5* exon 4 splicing profile in breast cancer cells (Supplementary Figure S7C,D). Collectively, our results indicate SRSF1 as a novel regulator of *DCUN1D5* exon 4 splicing.

## 4. Discussions

Breast cancer is one of the most common cancer types among women, affecting 2.1 million women each year (https://www.who.int/cancer/prevention/diagnosis-screening/breast-cancer/en/; accessed on 9 January 2021). Despite remarkable progress, our understanding of the molecular mechanisms involved in the development and progression of this tumor is still limited. In this study, we performed a highly specific, quantitative, and cost-effective RASL-seq of high- and low-metastatic breast cancer cells in order to identify AS programs that could characterize very aggressive breast cancer tumors. Our RASL-seq was able to detect 5530 annotated AS events. We found that 925 AS events, mainly affecting protein-coding genes, were altered in high- compared to low-metastatic cells, thus supporting a widespread role of AS in generating the proteomic diversity contributing to breast cancer progression. Moreover, our RT-PCR analysis, confirmed that RASL-seq shows a highly accurate and reproducible validation rate (90%, 18/20). Since RASL-seq provides an accurate and meaningful comparison of AS events between two groups, we are able to detect metastatic-specific AS without fruitless efforts.

GO analysis of differentially spliced genes showed enrichment for biological processes including apoptosis, Wnt signaling, cell cycle, DNA replication, cell–cell adhesion, RNA processing, and chromatin modifications. These results suggest that perturbation of AS profiles of these genes could play important roles in different aspects of breast cancer progression. Importantly, whereas up- and down-regulated AS cassette exons shared common biological pathways such as cell–cell adhesion, mRNA processing, positive regulation of GTPase activity, and apoptosis, biological processes specific to each category were also found.

Cassette exons differentially spliced between high- and low-metastatic breast cancer cells showed enrichment for a number of RNA motifs, which could represent binding sites for RBPs involved in breast cancer malignancy, including PTBP1 [54,55], SRSF1 [28,30], and SRSF9 [30].

Global analyses based on ESTs, whole genome-seq, and RNA-seq have been shown that breast cancers are characterized by numerous aberrant AS events [7,11,12,14,23,66,67,68,69,70]. Accordingly, our RASL-seq results identified a large number of AS events that were altered in high-metastatic breast cancer cells compared to low-metastatic breast cancer cells using the MDA-MB-231/MCF7 in vitro model for TNBC/ER positive breast tumors [71]. Notably, among AS events differentially expressed in MDA-MB-231 vs. MCF7 cells we found genes previously annotated as alternatively spliced in breast tumor tissues and between different breast cancer subtypes [27]. In particular, we found genes with key roles in breast oncogenesis and progression (for example *RAC1*, *MAP3K*, and *CTNND1*).

However, since MDA-MB-231 and MCF7 cells originated from different patients, we cannot exclude the possibility of individual differences in AS events. Nevertheless, we were able to confirm a high percentage (16/18) of aberrant AS events associated with high-metastatic MDA-MB-231 in another cellular model of low- and high-metastatic breast cancer cell lines (T47D vs. BT-549).

Furthermore, our analysis of annotated CCLE transcripts in 46 breast cancer cell lines clearly indicates that a large fraction of our identified AS changes are associated with the most aggressive and highly metastatic “triple-negative” breast cancer subtype.

Among differentially regulated AS cassette exons identified by our RASL-seq analysis, we found *Defect in cullin neddylation 1 domain containing 5* (*DCUN1D5*, a.k.a., squamous cell carcinoma related oncogene 5, SCCRO5) exon 4. *DCUN1D5* is a key component of the neddylation pathway, a post-translational modification involved in a number of biological processes such cell cycle progression, metabolism, immunity, and tumorigenesis [72]. Notably, it has been reported that *DCUN1D5* has oncogenic activity [35,58]. We found that *DCUN1D5* exon 4 was preferentially included in high metastatic breast cancer cells. We also demonstrated that an AS-NMD program, which occurs at the level of *DCUN1D5* exon 4, regulates the *DCUN1D5* steady-state mRNA levels. Accordingly, *DCUN1D5* expression was increased in high-metastatic compared to non-metastatic tumor tissues. Importantly, the oncogenic potential of *DCUN1D5* has been reported in two papers [35,58], showing that increased expression of *DCUN1D5* promoted migration, invasion, and transformation. Consistent with these results, we found that *DCUN1D5* showed higher expression in breast tumors than in normal breast tissues. Consistently, breast cancer patients in the high *DCUN1D5* expression group demonstrated a lower 5-year overall survival rate than those in the lower expression group.

To gain insights into the molecular mechanisms underlying *DCUN1D5* splicing, we focused on SRSF1 since binding motifs for this AS regulator are located within exon 4. Remarkably, we found that SRSF1 stimulated *DCUN1D5* exon 4 inclusion. SRSF1 is a prototypical member of the SR family and a proto-oncogene, whose expression levels are increased in different tumor types probably as a consequence of gene amplification [65,73,74,75]. Importantly, SRSF1 is a direct target of the proto-oncogene c-Myc and SRSF1 overexpression promotes tumor formation in mice [65,75]. SRSF1-mediated AS regulation generates protein isoforms promoting cell migration and EMT [7,52], which have pro-oncogenic capabilities or lack tumor suppressor activity [65,76,77], and influence angiogenesis [78]. Our data add *DCUN1D5* exon 4 to the list of SRSF1 splicing targets. Moreover, they support the conclusion that SRSF1 activation could contribute to the malignant progression of breast cancers by stabilizing *DCUN1D5* expression through the involvement of the AS-NMD pathway. Due to the relevance of *DCUN1D5* expression in metastatic breast cancer, a better characterization of *DCUN1D5* activity and its association with neddylation post-translation modifications will provide novel insights into breast cancer biology.

Aberrant AS has emerged as a key feature of breast cancer [11,12]. However, characterization of the functional roles for the majority of AS events associated to highly metastatic breast cancer cells is very limited. RASL-seq represents a rapid and cost-effective tool allowing the identification of AS programs associated with metastatic potential of breast cancer cells. In this regard, our work contributes to revealing AS switches associated with breast cancer metastasis that have the potential to serve as novel tools for diagnostic, prognostic, or therapeutic applications for breast cancer patients. Here, we focused on *DCUN1D5* splicing, however, future studies should understand how other identified AS events impact breast cancer progression and metastasis formation.

## Data Availability

All patient’s survival data that support findings of this study are available at GDC Data Portal (https://portal.gdc.cancer.gov/projects/TCGA-BRCA; accessed on 9 April 2021). Additional datasets retrieved by Oncomine or HCMDB include: GSE10893, GSE3521, GSE3744, GSE1477, GSE1477, and GSE22358.

## Supplementary Materials

The following are available online at https://www.mdpi.com/article/10.3390/cells10040858/s1, Figure S1: Validation of AS cassette exons up (**A**–**I**)- or down (**J**–**R**) -regulated in T47D vs. BT-549 breast cancer cell lines. (Left) For each gene, the schematic representation of the genomic region containing the AS cassette exon is shown. Lengths of cassette exons are shown in the green box. Inclusions of cassette exons are shown with dotted green lines. Skipping events are shown with dotted black lines. Primers used in RT-PCR are shown with arrows. (Right) RT-PCR analysis of the AS profile of cassette exons was performed by using RNA extracted from T47D and BT-549, Figure S2: (**A**–**I**) Percentage of RASL-seq selected cassette exon inclusion in 46 breast cancer cell lines from the CCLE database of events up-regulated in metastatic MDA-MB-231. (**J**–**R**) Percentage of cassette exon inclusion in 46 breast cancer cell lines from the CCLE database of events down-regulated in metastatic MDA-MB-231. Yellow = luminal breast cancer cell lines; orange = HER2 positive cell lines; red = TNBC cell lines. *DCUN1D5* exon 4 inclusion is shown in Supplementary Figure S4A. Error bars = +/- SEM. one-way ANOVA * *p* < 0.05 *** *p* < 0.001, Figure S3: (**A**) Scheme of AS cassette exons. Exons are shown with boxes. Introns are shown with lines. AS cassette exon is shown with green box and “Alt”, flanking constitutive exons are shown with white boxes and “C1” and “C2”. (**B**) Box plots showing 5′ and 3′ splice-site strengths in up- (red), or down- (green) AS cassette exons in high-metastatic cells and non-differentially regulated (gray) exons. Outliers are discarded. (**C**) Box plots showing exon length, upstream intron length, and exon GC content of cassette exons in high-metastatic cells, Figure S4: (**A**) Left: percentage of *DCUN1D5* NMD+ transcripts (lacking exon 4) in 46 breast cancer cell lines from the CCLE database. Yellow = luminal breast cancer cell lines; orange = HER2 positive cell lines; red = TNBC cell lines. Right: quantification of the percentage of *DCUN1D5* NMD+ transcripts by pooling breast cancer cell lines accordingly to their classification. Error bars indicate Min and Max values. * *p* < 0.05 (**B**) Left: *DCUN1D5* expression (RPKM) in 46 breast cancer cell lines from the CCLE database. Yellow = luminal breast cancer cell lines; orange = HER2 positive cell lines; red = TNBC cell lines. Right: quantification of *DCUN1D5* expression (RPKM) by pooling breast cancer cell lines accordingly to their classification. Error bars indicate Min and Max values. * *p* < 0.05; ** *p* < 0.01. (**C**) Correlation between percentage of *DCUN1D5* NMD+ transcripts and *DCUN1D5* expression (RPKM) in 46 breast cancer cell lines from the CCLE database. Pearson coefficient is indicated. Linear regression is also shown, Figure S5: (**A**) RT-PCR analysis of *DCUN1D5* exon 4 splicing in HEK-293 cells untreated (-), treated with DMSO or cycloheximide (CHX) for 6 h. CHX is able to stabilize the NMD+ transcript of *DCUN1D5.* (**B**) RT-qPCR analysis of total *DCUN1D5* mRNA expression levels (relative to *RPLP0*) in the same cells. (**C**) *DCUN1D5* splicing and (**D**) total *DCUN1D5* mRNA expression levels (relative to *RPLP0*) in HeLa cells untreated (-), treated with DMSO or with CHX for 6 h. Error bars indicate standard deviation (SD) calculated from three independent experiments. * *p* < 0.05, Figure S6: (**A**) Immunoblotting against GFP of MCF7 cells transfected with a plasmid expressing the DCUN1D5 GFP-tagged cDNA or the control (EGFP-C1) vector. α-VINCULIN as loading control. (**B**) Epifluorescent images of DCUN1D5-GFP (upper) or GFP (lower) overexpressing MCF7 cells. White arrows indicate the DCUN1D5 nuclear localization. Merge of GFP and DAPI signals is shown for each condition. Scale bar = 5 μm. (**C**) Cell proliferation assay of MCF7 cells untransfected, transfected with the control (EGFP-C1) or DCUN1D5-GFP vectors. Cell proliferation was analyzed 24 h upon transfection. N = 4. Anova one-way test. Errors bar indicate SD. ****p* < 0.001, Figure S7: (**A**) Immunoblotting against T7 of HEK-293 cells transfected with SRSF1-T7 or pcDNA 3.1 vectors. α-VINCULIN as loading control. (**B**) Left: RT-PCR analysis of *DCUN1D5* exon 4 splicing in SRSF1-T7 overexpressing cells. Right: quantification of DCUN1D5 exon 4 inclusion. Errors bar indicate SD. * *p* < 0.05, Table S1: List of primers used for RT-PCR, RT-qPCR and cloning, Table S2: List of up-: down- and non-differentially regulated AS events of genes in high-metastatic breast cancer cells. List of genes in each category shown in Figure 1A, Table S3: List of genes shown in Figure 1C,D, Table S4: List of breast cancer cell lines and Ensembl transcripts with or without the RASL-seq validated cassette exon shown in Supplementary Figure S2, Table S5: List of RBPs that potentially interact with RNA sequence motifs, Table S6: SRSF1 binding motifs predicted with ESEFinder 3.0, RBPMap, and SpliceAid2.
