# Supplementary material for: Widespread Alternative Splicing Changes in Metastatic Breast Cancer Cells
Source: Cells. 2021 Apr 9;10(4):858. doi: 10.3390/cells10040858 (PMC8070448; doi:10.3390/cells10040858)
Supplement: Supplementary file 1 [file cells-10-00858-s001.zip › Supplementary figures.pptx]

## Slide 1
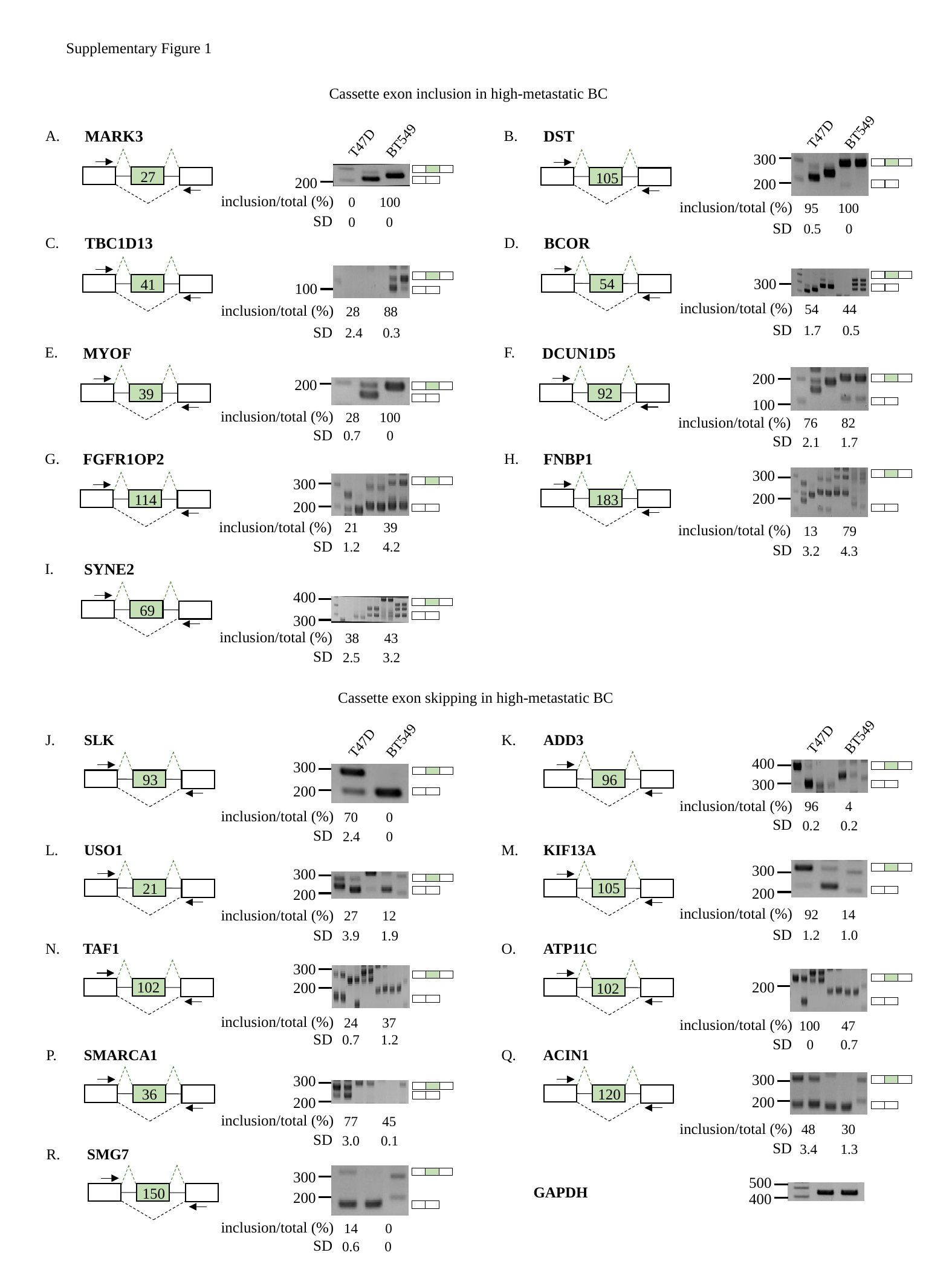

Supplementary Figure 1
Cassette exon inclusion in high-metastatic BC
BT549
T47D
A.
MARK3
27
B.
DST
105
BT549
T47D
300
200
200
inclusion/total (%)
0
100
inclusion/total (%)
95
100
SD
0
0
SD
0.5
0
C.
TBC1D13
41
D.
BCOR
54
300
100
inclusion/total (%)
54
44
inclusion/total (%)
28
88
SD
1.7
0.5
SD
2.4
0.3
E.
MYOF
39
F.
DCUN1D5
92
200
200
100
inclusion/total (%)
28
100
inclusion/total (%)
76
82
SD
0.7
0
SD
2.1
1.7
G.
FGFR1OP2
114
H.
FNBP1
183
300
300
200
200
inclusion/total (%)
21
39
inclusion/total (%)
13
79
SD
1.2
4.2
SD
3.2
4.3
I.
SYNE2
69
400
300
inclusion/total (%)
38
43
SD
2.5
3.2
Cassette exon skipping in high-metastatic BC
J.
SLK
93
BT549
T47D
300
200
inclusion/total (%)
70
0
BT549
K.
ADD3
96
T47D
400
300
inclusion/total (%)
96
4
SD
0.2
0.2
SD
2.4
0
L.
USO1
21
300
200
inclusion/total (%)
27
12
M.
KIF13A
105
300
200
inclusion/total (%)
92
14
SD
1.2
1.0
SD
3.9
1.9
N.
TAF1
102
300
200
inclusion/total (%)
24
37
O.
ATP11C
102
200
inclusion/total (%)
100
47
SD
0.7
1.2
SD
0
0.7
P.
SMARCA1
36
300
200
inclusion/total (%)
77
45
Q.
ACIN1
120
300
200
inclusion/total (%)
48
30
SD
3.0
0.1
SD
3.4
1.3
R.
SMG7
150
300
200
inclusion/total (%)
14
0
500
GAPDH
400
SD
0.6
0

## Slide 2
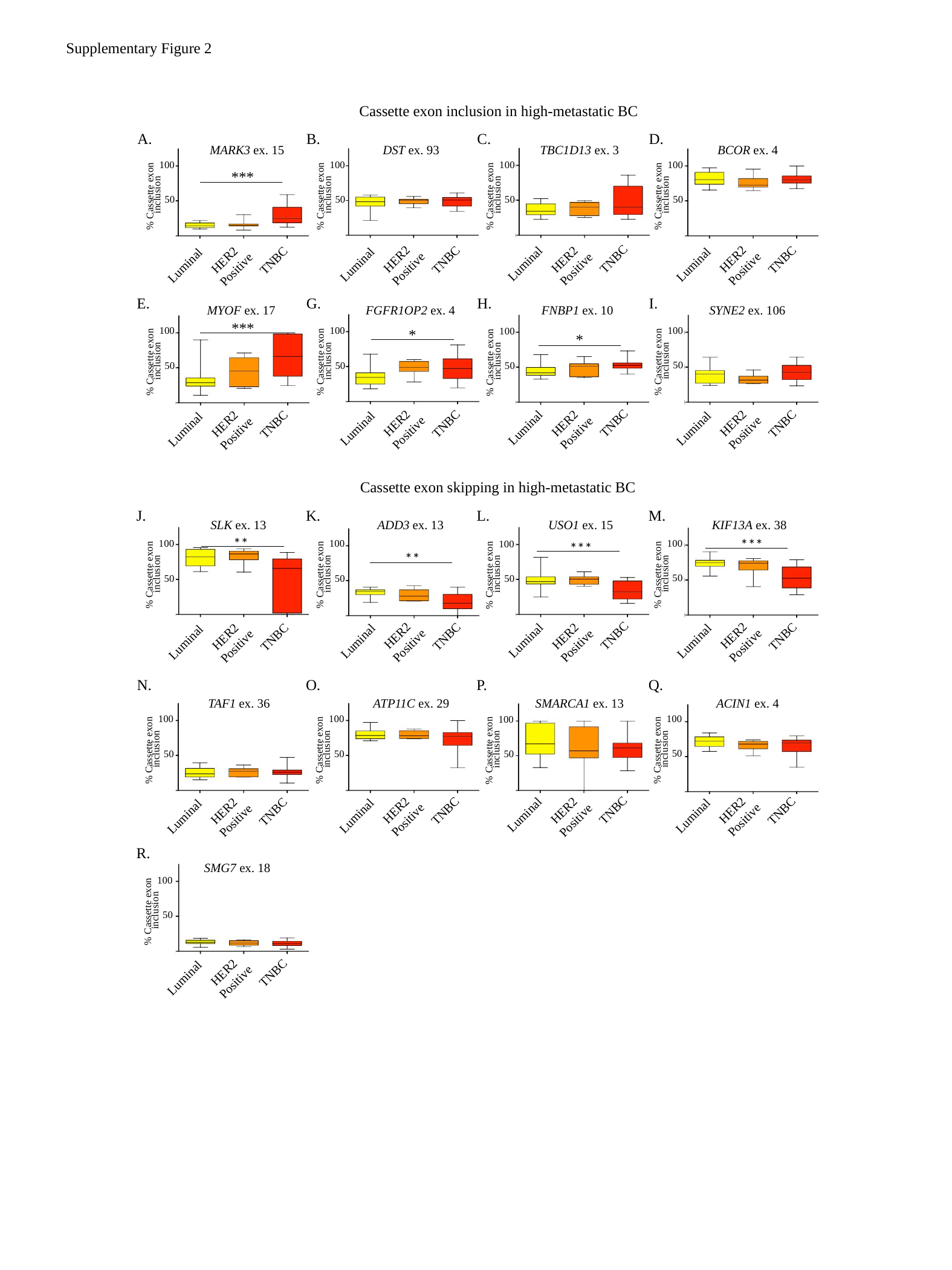

Supplementary Figure 2
Cassette exon inclusion in high-metastatic BC
A.
B.
C.
D.
MARK3 ex. 15
DST ex. 93
TBC1D13 ex. 3
BCOR ex. 4
100
100
100
100
***
% Cassette exon
inclusion
% Cassette exon
inclusion
% Cassette exon
inclusion
% Cassette exon
inclusion
50
50
50
50
HER2
 Positive
HER2
 Positive
HER2
 Positive
HER2
 Positive
TNBC
TNBC
TNBC
TNBC
Luminal
Luminal
Luminal
Luminal
E.
G.
H.
I.
MYOF ex. 17
FGFR1OP2 ex. 4
FNBP1 ex. 10
SYNE2 ex. 106
***
100
100
100
100
*
*
% Cassette exon
inclusion
% Cassette exon
inclusion
% Cassette exon
inclusion
% Cassette exon
inclusion
50
50
50
50
HER2
 Positive
HER2
 Positive
HER2
 Positive
HER2
 Positive
TNBC
TNBC
TNBC
TNBC
Luminal
Luminal
Luminal
Luminal
Cassette exon skipping in high-metastatic BC
J.
K.
L.
M.
SLK ex. 13
ADD3 ex. 13
USO1 ex. 15
KIF13A ex. 38
**
***
100
100
100
100
***
**
% Cassette exon
inclusion
% Cassette exon
inclusion
% Cassette exon
inclusion
% Cassette exon
inclusion
50
50
50
50
HER2
 Positive
HER2
 Positive
HER2
 Positive
HER2
 Positive
TNBC
TNBC
TNBC
TNBC
Luminal
Luminal
Luminal
Luminal
N.
O.
P.
Q.
TAF1 ex. 36
ATP11C ex. 29
SMARCA1 ex. 13
ACIN1 ex. 4
100
100
100
100
% Cassette exon
inclusion
% Cassette exon
inclusion
% Cassette exon
inclusion
% Cassette exon
inclusion
50
50
50
50
HER2
 Positive
HER2
 Positive
HER2
 Positive
HER2
 Positive
TNBC
TNBC
TNBC
TNBC
Luminal
Luminal
Luminal
Luminal
R.
SMG7 ex. 18
100
% Cassette exon
inclusion
50
HER2
 Positive
TNBC
Luminal

## Slide 3
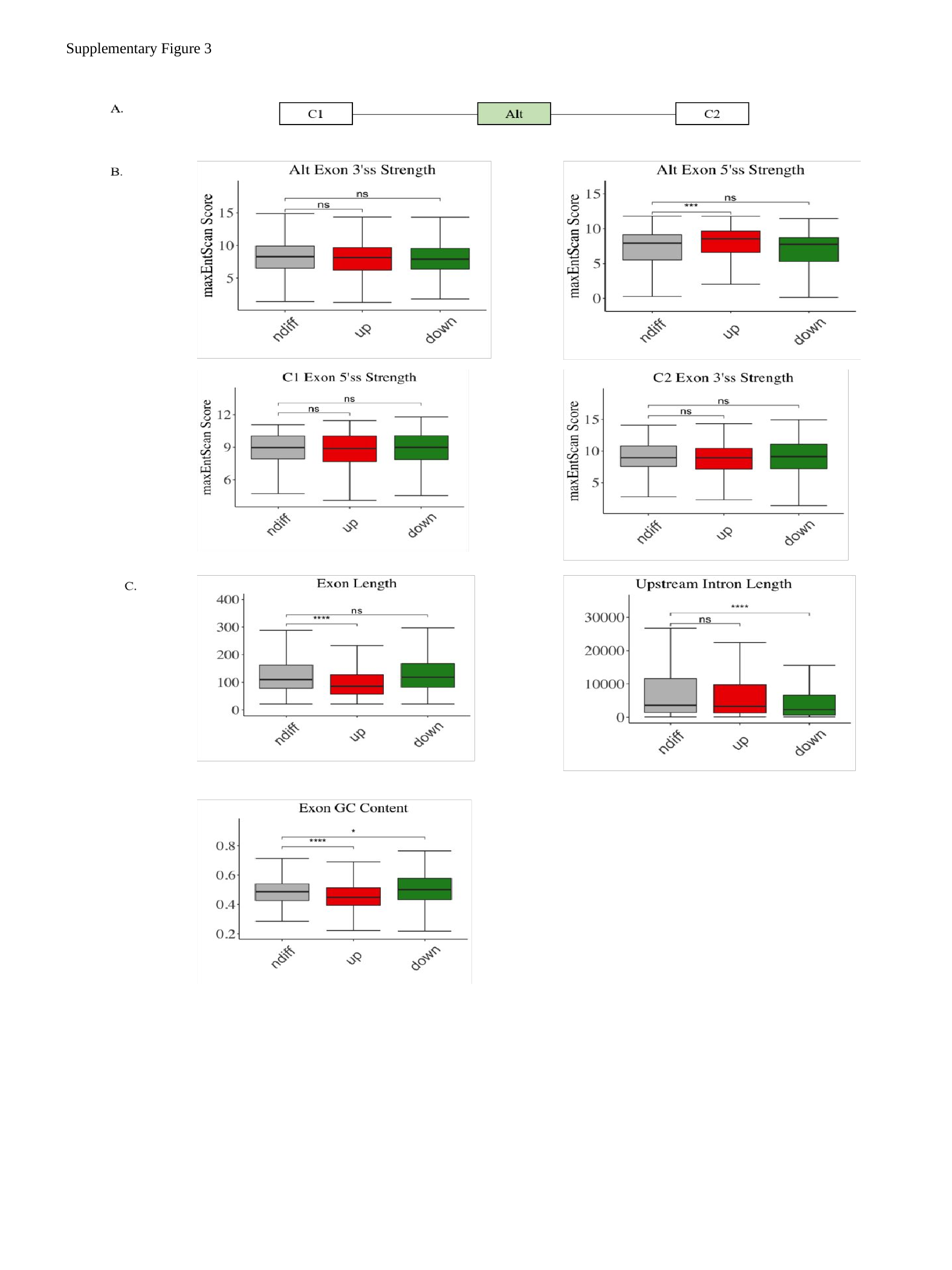

Supplementary Figure 3

## Slide 4
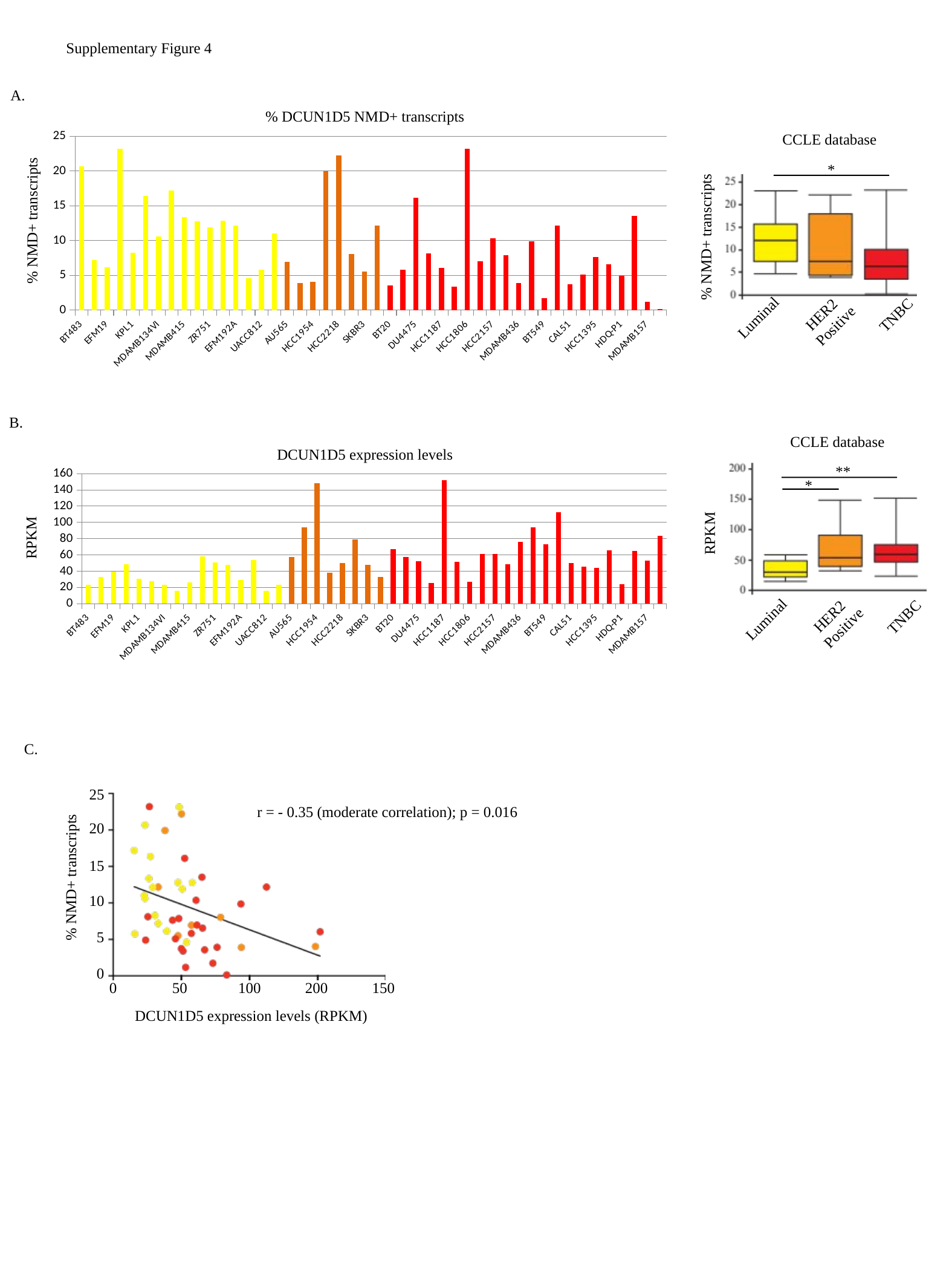

Supplementary Figure 4
A.
% DCUN1D5 NMD+ transcripts
### Chart
| Category | |
|---|---|
| BT483 | 20.68376068376068 |
| CAMA1 | 7.207752877044205 |
| EFM19 | 6.140573458513067 |
| HCC1428 | 23.14509480626545 |
| KPL1 | 8.276311502117949 |
| MCF7 | 16.36893911775429 |
| MDAMB134VI | 10.60735671514115 |
| MDAMB175VII | 17.18547341115434 |
| MDAMB415 | 13.34349980937857 |
| T47D | 12.79089812101362 |
| ZR751 | 11.89913317572892 |
| BT474 | 12.80487804878049 |
| EFM192A | 12.13058419243986 |
| MDAMB361 | 4.604530263646458 |
| UACC812 | 5.768025078369891 |
| ZR7530 | 11.01843722563652 |
| AU565 | 6.954102920723226 |
| HCC1569 | 3.902179691653376 |
| HCC1954 | 4.023724472602279 |
| HCC202 | 19.92648989236005 |
| HCC2218 | 22.20673172674766 |
| MDAMB453 | 8.040583386176287 |
| SKBR3 | 5.50304557865993 |
| UACC893 | 12.17075386012715 |
| BT20 | 3.574620196604111 |
| CAL148 | 5.833188229148527 |
| DU4475 | 16.10444063274252 |
| HCC1143 | 8.124018838304549 |
| HCC1187 | 6.059408549035088 |
| HCC1599 | 3.409312293006039 |
| HCC1806 | 23.19548872180451 |
| HCC1937 | 6.976744186046513 |
| HCC2157 | 10.3646517739816 |
| HCC70 | 7.864702220377667 |
| MDAMB436 | 3.931847968545215 |
| MDAMB468 | 9.86231187960295 |
| BT549 | 1.735684023506902 |
| CAL120 | 12.17105263157895 |
| CAL51 | 3.734025559105432 |
| CAL851 | 5.095123551279245 |
| HCC1395 | 7.627118644067767 |
| HCC38 | 6.556877096675816 |
| HDQ-P1 | 4.91596638655462 |
| Hs578T | 13.52553289372795 |
| MDAMB157 | 1.185547610086564 |
| MDAMB231 | 0.14405762304922 |CCLE database
*
% NMD+ transcripts
% NMD+ transcripts
Luminal
HER2
 Positive
TNBC
B.
CCLE database
DCUN1D5 expression levels
**
### Chart
| Category | |
|---|---|
| BT483 | 23.4 |
| CAMA1 | 33.02 |
| EFM19 | 39.41 |
| HCC1428 | 48.52 |
| KPL1 | 30.69 |
| MCF7 | 27.43 |
| MDAMB134VI | 23.38 |
| MDAMB175VII | 15.42 |
| MDAMB415 | 26.23 |
| T47D | 58.01 |
| ZR751 | 50.76000000000001 |
| BT474 | 47.56 |
| EFM192A | 29.1 |
| MDAMB361 | 53.86 |
| UACC812 | 15.95 |
| ZR7530 | 22.78 |
| AU565 | 57.52 |
| HCC1569 | 94.05 |
| HCC1954 | 148.37 |
| HCC202 | 38.09 |
| HCC2218 | 50.21 |
| MDAMB453 | 78.85 |
| SKBR3 | 47.61 |
| UACC893 | 33.03 |
| BT20 | 67.14 |
| CAL148 | 57.43 |
| DU4475 | 52.47000000000001 |
| HCC1143 | 25.48 |
| HCC1187 | 151.83 |
| HCC1599 | 51.33 |
| HCC1806 | 26.6 |
| HCC1937 | 61.49 |
| HCC2157 | 60.88 |
| HCC70 | 48.19 |
| MDAMB436 | 76.3 |
| MDAMB468 | 93.68999999999998 |
| BT549 | 73.16999999999997 |
| CAL120 | 112.48 |
| CAL51 | 50.08 |
| CAL851 | 45.73000000000001 |
| HCC1395 | 43.66 |
| HCC38 | 65.58 |
| HDQ-P1 | 23.8 |
| Hs578T | 65.21 |
| MDAMB157 | 53.14000000000001 |
| MDAMB231 | 83.30000000000001 |*
RPKM
RPKM
Luminal
HER2
 Positive
TNBC
C.
25
r = - 0.35 (moderate correlation); p = 0.016
20
15
% NMD+ transcripts
10
5
0
0
50
100
200
150
DCUN1D5 expression levels (RPKM)

## Slide 5
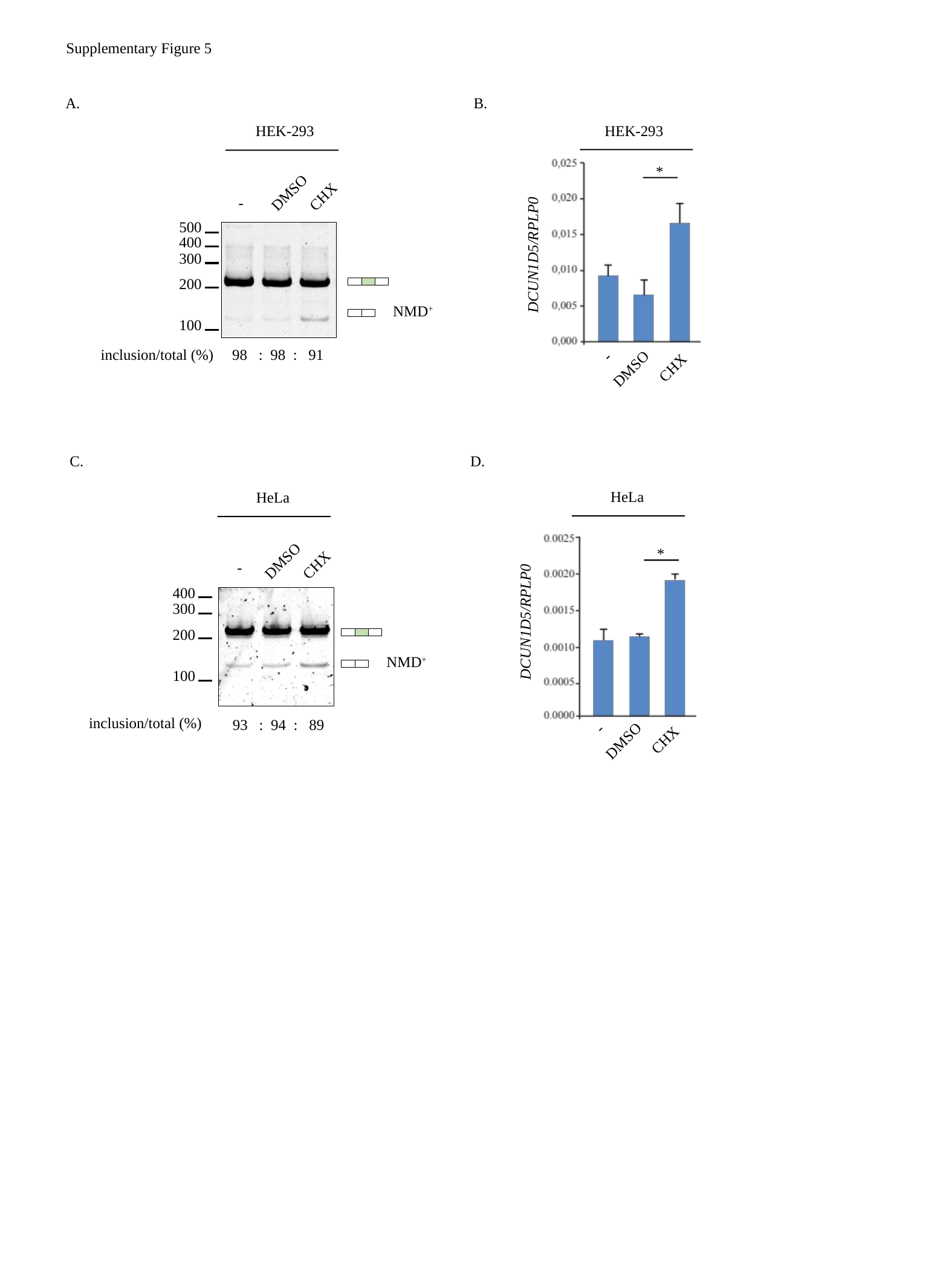

Supplementary Figure 5
A.
B.
HEK-293
HEK-293
*
DMSO
CHX
-
500
400
DCUN1D5/RPLP0
300
200
NMD+
100
-
98 : 98 : 91
inclusion/total (%)
DMSO
CHX
C.
D.
HeLa
HeLa
DMSO
CHX
*
-
400
300
DCUN1D5/RPLP0
200
NMD+
100
inclusion/total (%)
93 : 94 : 89
-
DMSO
CHX

## Slide 6
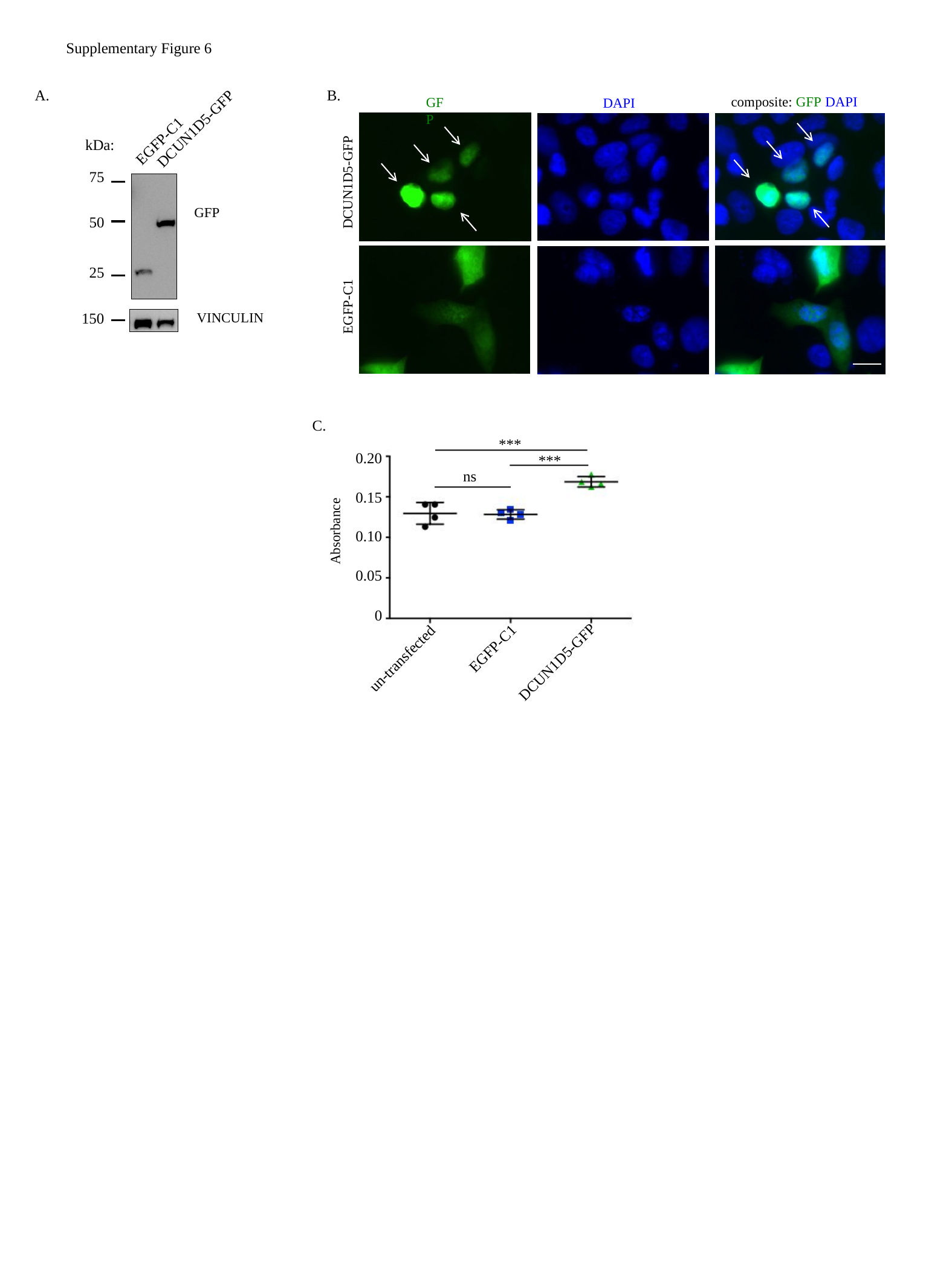

Supplementary Figure 6
A.
B.
composite: GFP/DAPI
GFP
DAPI
DCUN1D5-GFP
EGFP-C1
kDa:
75
DCUN1D5-GFP
GFP
50
25
EGFP-C1
VINCULIN
150
C.
***
0.20
***
ns
0.15
Absorbance
0.10
0.05
0
EGFP-C1
un-transfected
DCUN1D5-GFP

## Slide 7
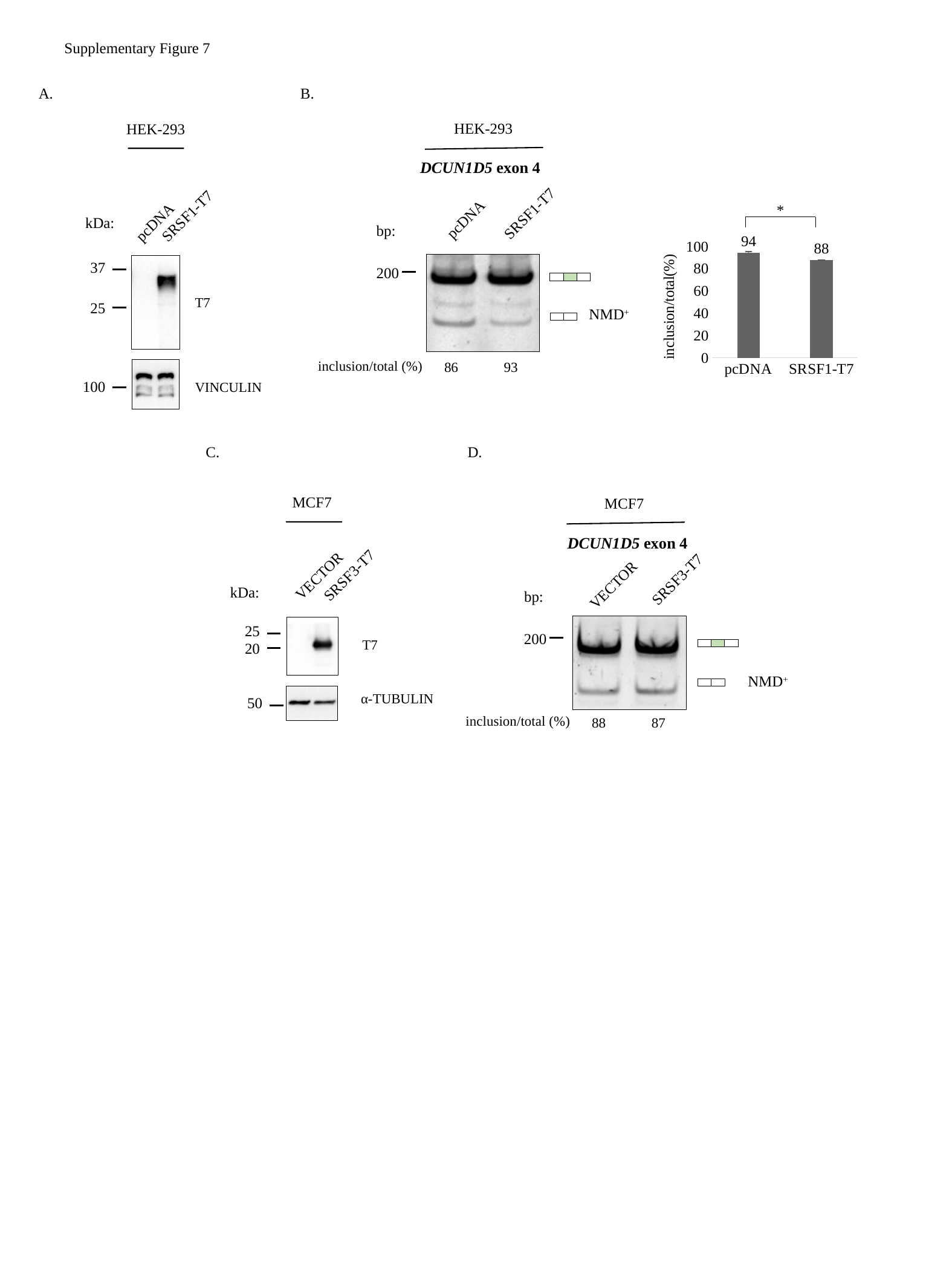

Supplementary Figure 7
A.
B.
HEK-293
HEK-293
DCUN1D5 exon 4
*
### Chart
| Category | |
|---|---|
| pcDNA | 94.21 |
| SRSF1-T7 | 87.78 |SRSF1-T7
SRSF1-T7
pcDNA
pcDNA
kDa:
bp:
37
200
T7
25
NMD+
inclusion/total (%)
86
93
100
VINCULIN
C.
D.
MCF7
MCF7
DCUN1D5 exon 4
SRSF3-T7
VECTOR
SRSF3-T7
VECTOR
kDa:
bp:
25
200
T7
20
NMD+
α-TUBULIN
50
inclusion/total (%)
88
87
